# Irx1 mechanisms for oral epithelial basal stem cell plasticity during reepithelialization after injury

**DOI:** 10.1172/jci.insight.179815

**Published:** 2025-01-09

**Authors:** Dan Su, Tadkamol Krongbaramee, Samuel Swearson, Yan Sweat, Mason Sweat, Fan Shao, Steven Eliason, Brad A. Amendt

**Affiliations:** 1 Department of Anatomy and Cell Biology,; 2 Craniofacial Anomalies Research Center, Carver College of Medicine, and; 3 Iowa Institute for Oral Health Research, College of Dentistry, The University of Iowa, Iowa City, Iowa, USA.; 4 Division of Endodontics, Department of Restorative Dentistry & Periodontology, Faculty of Dentistry, Chiang Mai University, Chiang Mai, Thailand.; 5 Harvard University, Boston, Massachusetts, USA.

**Keywords:** Cell biology, Stem cells, Adult stem cells, Cell migration/adhesion, Human stem cells

## Abstract

The oral mucosa undergoes daily insults, and stem cells in the epithelial basal cell layer regenerate gingiva tissue to maintain oral health. The Iroquois Homeobox 1 (IRX1) protein is expressed in the stem cell niches in human/mouse oral epithelium and mesenchyme under homeostasis. We found that *Irx1^+/–^* heterozygous (Het) mice have delayed wound closure, delayed morphological changes of regenerated epithelium, and defective keratinocyte proliferation and differentiation during wound healing. RNA-Seq analyses between WT and *Irx1^+/–^* mice at 3 days postinjury (dpi) found impaired epithelial migration and decreased keratinocyte-related genes upon injury. IRX1-expressing cells are found in the gingival epithelial basal cell layer, a stem cell niche for gingival maintenance. IRX1-expressing cells are also found in cell niches in the underlying stroma. IRX1 activates SOX9 in the transient amplifying layer to increase cell proliferation, and EGF signaling is activated to induce cell migration. *Krt14^CreERT^* lineage tracing experiments reveal defects in the stratification of the *Irx1^+/–^* HET mouse oral epithelium. IRX1 is primed at the base of the gingiva in the basal cell layer of the oral epithelium, facilitating rapid and scarless wound healing through activating SOX9 and the EGF signaling pathway.

## Abstract 2

The increasing prevalence of chronic wounding and scarring, which affects people’s quality of life and increases healthcare costs has become more prominent. Thus, identifying the oral epithelial stem cell (OESC) factors that contribute to gingival wound healing can also provide insights to improve oral and dental health.

## Graphical abstract

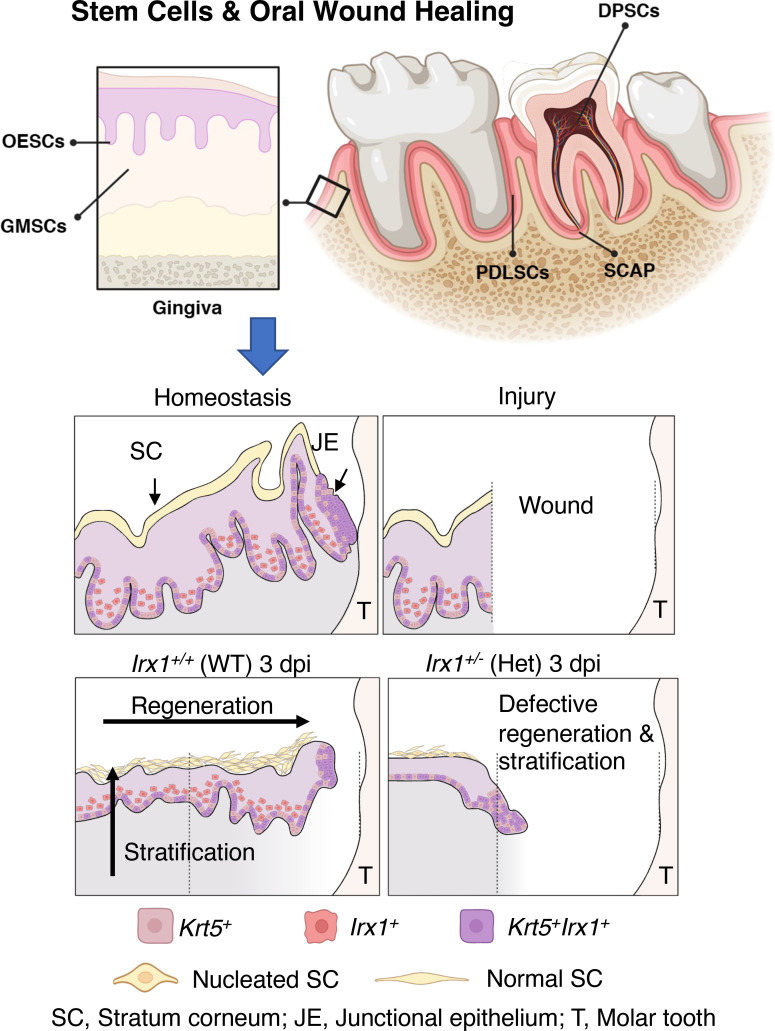

## Introduction

Gingiva is the soft tissue surrounding the teeth that functions to protect the dental and periodontal tissue in the deeper layers from damage and infection. It is composed of the gingival epithelium (GE) and the underlying lamina propria. The attached GE is a keratinized stratified epithelium in the oral cavity, consisting of 4 different layers, including the basal, spinous, granular, and stratum corneum layers, which largely resemble the structure of the epidermis (1). Moreover, injured gum tissue undergoes a wound healing process similar to that of the skin, though it is more rapid and scarless. This feature of efficient wound healing in the gingiva makes it a great model to study optimal wound resolution (2). Thus, it becomes increasingly important to identify the factors and regulatory networks in gingival wound healing to gain insights into the improvement of wound healing process to meet the dramatically increasing need for treating deficient healings (3).

The general wound healing process is composed of 3 distinct yet overlapped stages: (a) inflammation stage; (b) new tissue formation stage, and (c) tissue remodeling stage (2, 4). A number of activities take place during the new tissue formation stage, including reepithelization, differentiation of myofibroblasts, and the formation of new capillaries into the wound bed. The regulation of keratinocyte (KC) activities is the major contributor to reepithelization, involving the proliferation, migration, and differentiation of basal cells (2, 4). Growth factors and signaling pathways are highly involved in KC regulation. For example, epidermal growth factor (EGF) signaling plays an essential role in regulating KC proliferation, migration, survival, and epithelial-mesenchymal transition (5, 6). While the cellular and molecular regulation of cutaneous wound healing processes have been well documented, there is a limited understanding of the rapid gingival wound healing process. It has been reported that a set of genes that are only activated upon wounding in the skin remain primed at a basal level in the oral mucosa to facilitate the wound healing process (7). *Sox2* and *Pitx1* are 2 of the primed genes in the homeostatic oral mucosa, which positively regulate KC migration and proliferation (7, 8).

Iroquois genes encode 2 clusters of *Irx* genes, located on mouse chromosome 8 and 13, and function in tissue differentiation in mammals (9). Among the genes from this family, Iroquois Homeobox 1 (IRX1) is expressed in the developing mammalian organs and regulates cell differentiation in tooth, lung, kidney, limbs, heart, blood cells, and neural cells (10–19). We previously reported that IRX1 was expressed as a regulator of the differentiation of dental epithelial stem cells as well as lung progenitor cells during embryogenesis (10). However, limited research has been done to unravel the role of *Irx1* in maintaining homeostasis and regeneration of adult tissues. We have found that IRX1 is expressed in the basal cells of the epithelium and in cell colonies in the lamina propria of human gingival tissue. The expression of IRX1 in the basal cells of adult gingival tissue suggests a potential role for IRX1 in the maintenance of basal cell homeostasis and regulation of basal cells in response to gingival injury.

In this study, we have found that IRX1 is expressed in the basal and immediate suprabasal cells of the epithelium and in colonies or niches of the lamina propria in human and murine gingival tissue. We establish a mouse gingival injury model to examine the function of IRX1 in epithelial basal cells during gingival wound healing. Haploinsufficiency of *Irx1* in adult mice negatively affected reepithelialization during gingival wound healing, resulting in delayed wound closure, morphological defects in regenerated epithelium (RE), and altered KC differentiation. Lineage tracing and label retaining cells of the oral epithelium revealed defects in reepithelialization and stratification of the gingiva. Transcriptomic analysis revealed that the wound-activated signature was diminished in the *Irx1^+/–^* RE compared with the *Irx1^+/+^* RE at 3 days postinjury (dpi). *Sox9*, a direct target of IRX1 in the epithelium, and altered EPGN expression, a ligand for EGF signaling pathway expressed in the KCs, may be responsible for the phenotype in the *Irx1^+/–^* RE.

## Results

### IRX1 is expressed in the basal stem cell layer of murine and human gingiva and a potential mesenchymal stroma cell niche.

**Figure 1:**
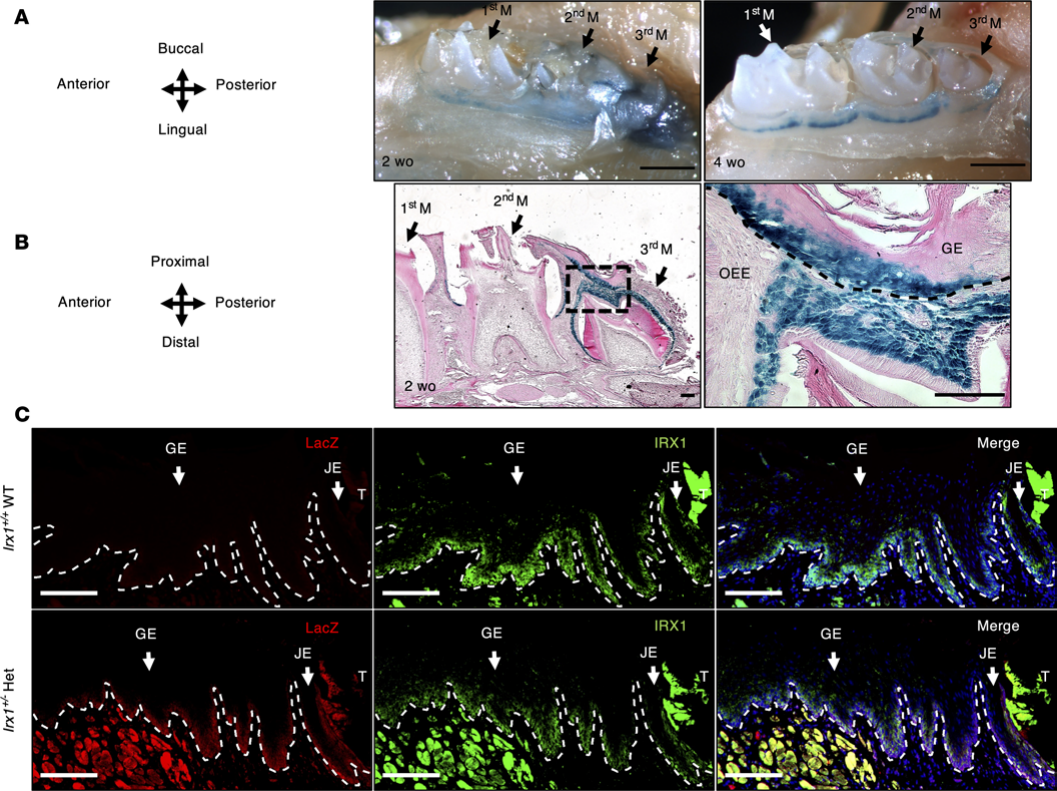
IRX1 is expressed in the basal layer of mouse gingiva. (**A**) Whole mount X-gal staining (blue, IRX1 expression) in mouse mandible in *Irx1^+/–^* adult mice at 2 or 4 weeks old (wo). Scale bar: 500 μm. (**B**) Eosin staining of sections from X-gal–stained adult mandible at 2 wo. Scale bar: 100 μm. (**C**) Representative IF staining of IRX1 and LacZ in *Irx1^+/+^* and *Irx1^+/–^* 3-month-old gingiva area in front of the first molar. Blue staining represents nuclei. Scale bar: 100 μm. M, molar; OEE, outer enamel epithelium; T, tooth; GE, gingival epithelium; JE, junctional epithelium.

To analyze IRX1 expression in the oral epithelial basal layer in mice, we first performed whole-mount X-gal staining of mouse mandibular tissue from our recently generated heterozygous (*Irx1^+/–^*; HET) mouse strain, in which exons 1–4 (the entire coding sequence) on 1 allele of the endogenous *Irx1* gene were replaced by a *LacZ* reporter gene and neomycin resistance gene (10). We found that IRX1 is expressed in the gingiva around the molar rows (Figure 1A). Sections from the X-gal–stained tissue showed that IRX1 was expressed in the basal and immediate suprabasal layer of the GE, as well as in the outer enamel epithelium (OEE) (Figure 1B). Immunofluorescence (IF) staining of adult mice showed that IRX1 was expressed in the junctional epithelium (JE) and the basal layer and immediate suprabasal layer of the gingiva in front of the first molar. IRX1 antibody staining overlapped with X-gal staining in the *Irx1^+/–^* samples, indicating the specificity of the IRX1 antibody. The expression level of IRX1 was reduced in the *Irx1^+/–^* Het gingiva under homeostasis due to the deletion of 1 allele, but the expression pattern did not change compared with WT (Figure 1C).

In addition, we analyzed 2 deidentified human gingiva samples for IRX1 expression and found that IRX1 was expressed in the basal and immediate suprabasal layer of the epithelium as well as in a stroma mesenchymal cell niche (Supplemental Figure 1, A–F; supplemental material available online with this article; https://doi.org/10.1172/jci.insight.179815DS1). As a control, H&E-stained human gingival samples are shown depicting normal histology (Supplemental Figure 2). To initially determine if these mesenchymal cells were contained in a stem cell niche, we costained with STRO-1, a marker for mesenchymal stem cells (20, 21). Both IRX1 and STRO-1 were coexpressed in these cells, suggesting a possible stem cell niche in the mesenchymal stromal layer (Supplemental Figure 1G).

Because GE is a keratinized stratified epithelium similar to the skin epidermis (1), we asked if IRX1 had a similar expression pattern in the skin epithelium layer. Interestingly, we found that IRX1 was not expressed in the epidermis but was instead expressed in the hair follicles by performing X-gal and eosin staining of the skin tissue on the back of *Irx1^+/–^* mice at different ages (Supplemental Figure 3, A–D). In addition, IRX1 is expressed in the bulge and dermal papilla regions of the mature hair follicles, where stem cells reside contributing to skin homeostasis and the healing of cutaneous wounds (22–24). JE and the basal layer of GE serve as pools of gingival epithelial stem/progenitor cells (25–28). Considering that IRX1 is highly expressed in these regions, it suggests a potential role for IRX1 in stem/progenitor cell maintenance and tissue healing.

### Irx1 haploinsufficiency delays gingival wound closure and reepithelization.

**Figure 2:**
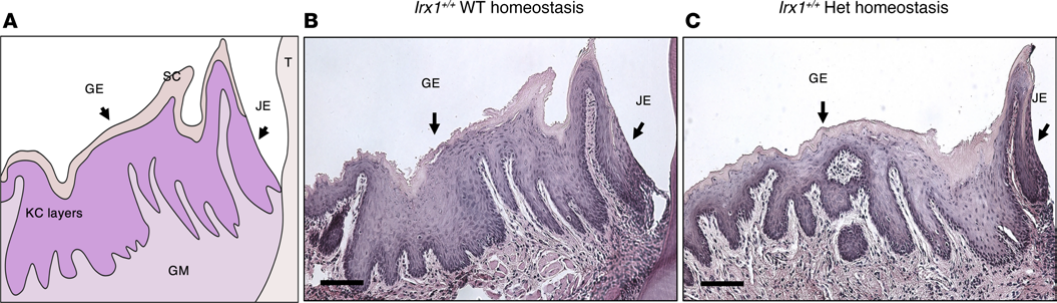
*Irx1^+/–^* mice show defective stratification of the mouse oral epithelium. (**A**) Schematics of the structure of the gingiva in front of the first molar. (**B**) Representative H&E staining of the homeostatic gingiva of a 3-month-old (mo) *Irx1^+/+^* mouse. Scale bar: 100 μm. (**C**) H&E staining of the homeostatic gingiva of 3 mo *Irx1^+/–^* mouse. Scale bar: 100 μm. T, tooth; GE, gingival epithelium; JE, junctional epithelium; SC, stratum corneum; KC, keratinocyte; GM, gingival mesenchyme.

We assessed the morphology of homeostatic tissue of *Irx1^+/+^* and *Irx1^+/–^* mice before examining the injured tissue. The morphology, thickness, and stratification of the adult gingiva in front of the first molar in *Irx1^+/–^* mice is thinner and less developed compared with *Irx1^+/+^* mice (Figure 2). These results suggest defects in oral epithelial stratification.

**Figure 3:**
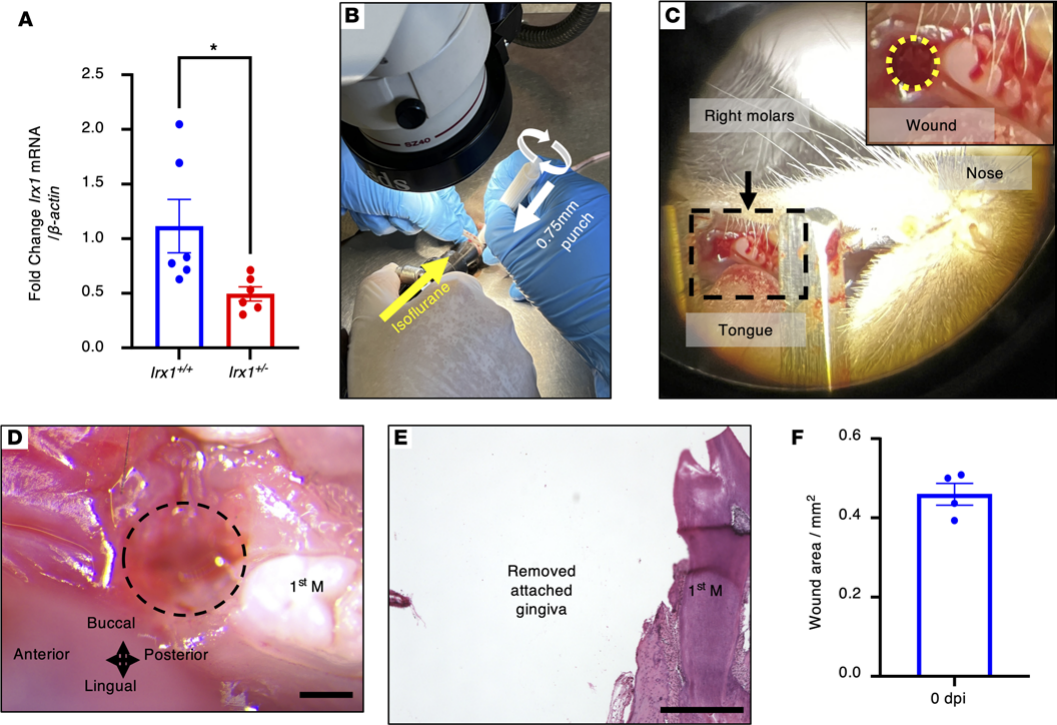
Establishment of a mouse gingival injury model. (**A**) The expression level of *Irx1* mRNA in *Irx1^+/+^* and *Irx1^+/–^* gingival tissues in front of the first molar assessed by qPCR. *n* = 6. (**B** and **C**) Operation technique for the mouse gingival injury model. Mice aged 10–16 weeks old were subjected to injury. (**B**) After anesthesia, the mouth was opened using a retractor and the wound was made with a biopsy punch (ø0.75 mm). (**C**) Picture taken from the dissection scope showing that the wound was made in front of the first molar on the right side. (**D** and **E**) The gingiva, including the junctional epithelium, was removed after the surgery. Scale bar: 500 μm. (**F**) Quantitation of the wound area. *n* = 4. M, molar.

Oral epithelium, including GE, is generally recognized as primed with a set of genes, whose expression facilitates the fast and scarless wound healing process compared with cutaneous wounds (7, 29–31). The expression pattern of IRX1 indicated it is one of the genes that contribute to gingival wound healing. To test this hypothesis, we established a murine gingival wound healing model in adult (10–16 weeks old [wo]) *Irx1^+/+^* WT and *Irx1^+/–^* (Het) mice, since the *Irx1^–/–^* null mice are early neonatal lethal (10). We extracted RNA from the unwounded gingival tissue in front of the first molar and performed quantitative PCR (qPCR). The homeostatic gingival tissue of *Irx1^+/–^* mice showed significantly lower expression of *Irx1* compared with *Irx1^+/+^* animals (*n* = 6) (Figure 3A). The gingival tissue in front of the first molar was removed with a biopsy punch (ø0.75 mm) (Figure 3B). Every wound was carefully confirmed under the dissection scope to ensure (a) the wound site was consistently in front of the right first molar and (b) the JE was removed (Figure 3C). To confirm the consistency of the operation, we sacrificed mice from both genotypes immediately after surgery and dissected the mandible for imaging under a dissection scope (Figure 3D). H&E staining of sagittal sections of the tissue further validated that JE and GE were both removed (Figure 3E). The wound size measurements indicated that a relatively consistent wound area (~0.45 mm^2^) was created in the mice (Figure 3F).

**Figure 4:**
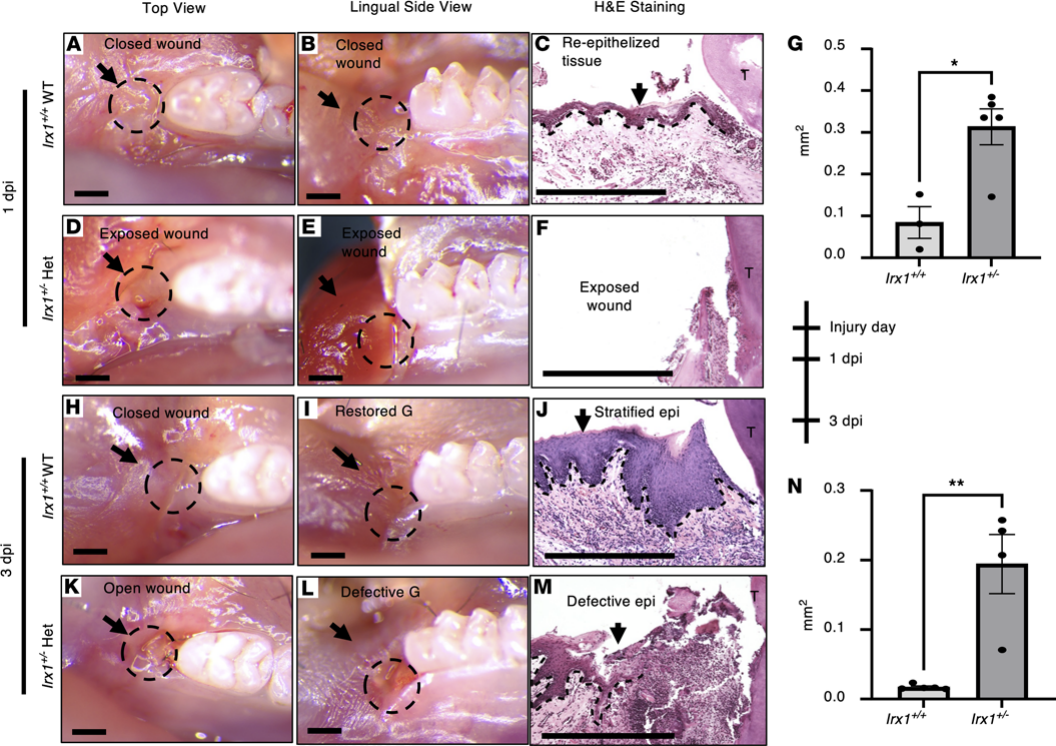
*Irx1^+/–^* mice exhibit delayed wound closure and reepithelization. Mouse mandible tissues were collected at 1 day postinjury (dpi) and 3 dpi. (**A**–**F**) Mandible tissue harvested at 1 dpi. (**A**–**C**) Representative mandible images and H&E staining show the healing of *Irx1^+/+^* mice at 1 dpi. The wound was closed, and a thin layer of epithelium was formed. (**D**–**F**) Representative mandible images and H&E staining show the healing of *Irx1^+/–^* mice at 1 dpi. The wound is largely exposed. (**G**) Quantitation of the wound size in *Irx1^+/+^* and *Irx1^+/–^* samples at 1 dpi. *n* = 3-5. (**H**–**M**) Mandible tissue harvested at 3 dpi. (**H**–**J**) Representative mandible images and H&E staining show the healing of *Irx1^+/+^* mice at 3 dpi. The wound was fully closed, and epithelium layer was stratified. (**K**–**M**) Representative mandible images and H&E staining show the healing of *Irx1^+/–^* mice at 3 dpi. The wound was still open, and reepithelization was not complete. (**N**) Quantitation of the wound size in *Irx1^+/+^* and *Irx1^+/–^* samples at 1 dpi. *n* = 4–5. Black dashed lines demarcate the gingival epithelium and mesenchyme. epi, epithelium; G, gingiva. Scale bar: 500 μm.

Next, we assessed oral epithelial regeneration activity in *Irx1*^+/+^ and *Irx1^+/–^* mice at 1 dpi and 3 dpi. At 1 dpi, wounds in *Irx1^+/+^* samples were almost closed, with an average remaining wound size of 0.08 mm^2^ (*n* = 3) (Figure 4, A, B, and G). H&E images show that the reepithelization was complete. However, the RE was not fully stratified and was absent of rete pegs, with immature formation of the JE (Figure 4C). These features indicate that the RE in *Irx1^+/+^* mice was in the process of tissue remodeling at 1 dpi. In *Irx1^+/–^* mice at 1 dpi, however, the wounds were largely open, with an average size of 0.31 mm^2^ (*n* = 5) (Figure 4, D, E, and G). The RE was absent in the 500 μm region proximal to the first molar in H&E staining, indicating that reepithelization was incomplete (Figure 4F).

Immune homeostasis is essential for tissue regeneration, and immune effector cells such as macrophages are mediators of tissue regeneration (32, 33). Furthermore, exosomes derived from mesenchymal cells affect wound healing, and both macrophages and exosomes are characterized by CD63 and CD11b expression (32, 33). The initial phase of wound healing involves the migration of macrophages and exosomes to the wound site to illicit the wound healing response. To determine if Irx1 played a role in the immune response, we analyzed the wound response for CD63 and CD11b expression at 1 dpi in both *Irx1^+/+^* and *Irx1^+/–^* mice. The expression of CD63 was unchanged in the wound area and mesenchyme of both *Irx1^+/+^* and *Irx1^+/–^* mice at 1 dpi (Supplemental Figure 4). The expression of CD11b was unchanged in both injured mice at 1 dpi (Supplemental Figure 5). These data suggest that *Irx1* does not regulate the immune response in oral wound healing.

At 3 dpi, wounds in the *Irx1^+/+^* samples exhibited partially restored gingiva (Figure 4, H and I). H&E staining showed the formation of stratified epithelium and rete pegs, but without the JE (Figure 4J). The average remaining wound size was 0.01 mm^2^ (*n* = 5) (Figure 4N). In the *Irx1^+/–^* mice, the wounds were open and reepithelization remained incomplete (Figure 4, K and L). The regenerative front (RF) was ~600 μm to the first molar (Figure 4M). The average remaining wound size was 0.19 mm^2^ at 3 dpi in *Irx1^+/–^* mice (*n* = 4) (Figure 4N). At 7dpi, the *Irx1^+/+^* mice had completely restored gingiva with fully developed GE and JE. However, the *Irx1^+/–^* mice had a remaining wound size of 0.02 mm^2^, and the development of the GE and JE was incomplete (*n* = 4) (Figure 4N). Together, these data show a delayed gingival wound closure and morphological recovery due to haploinsufficiency of *Irx1^+/‑^* after injury compared with WT mice. These data demonstrate a major role for *Irx1* in regulating gingival wound healing.

### KC differentiation in the RE is defective in Irx1^+/–^ HET mice at 3 dpi.

During wound healing, successful reepithelization requires the rigid orchestration of KC activities, including migration, proliferation and differentiation. The delay of reepithelization made us ask if KC activities were affected in the RE.

**Figure 5:**
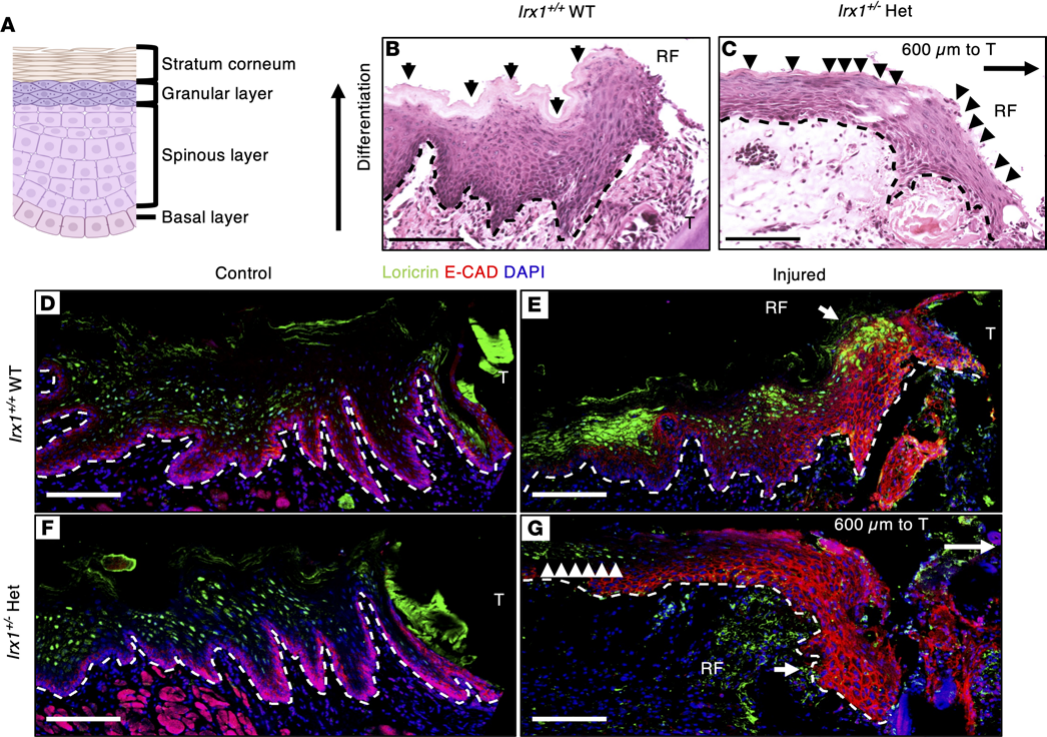
*Irx1^+/–^* mice show delayed keratinocyte differentiation in the RE at 3 dpi. (**A**) Schematics showing the structure of stratified epithelium in the gingiva. Keratinocytes originating from the basal layer differentiate and move vertically to become mature keratinocytes to form spinous layer and granular layer. The stratum corneum layer consists of dead keratinocytes, indicating a successful differentiation process. (**B** and **C**) Representative H&E staining images showing the regenerative front (RF) during reepithelization in *Irx1^+/+^* and *Irx1^+/–^* mice at 3 dpi. Scale bar: 100 μm. (**B**) The regenerated epithelium of *Irx1^+/+^* at 3 dpi. Arrows indicate the fully formed stratum corneum. (**C**) The regenerated epithelium of *Irx1^+/–^* at 3 dpi. Arrowheads indicate the nucleus-containing cells in the superficial layer of the regenerated epithelium. The regenerative front is ~600 μm to the first molar. The stratum corneum is absent at this stage in *Irx1^+/–^*. (**D**–**G**) Representative IF staining images of Loricrin and E-cadherin in control and injured gingival tissue of *Irx1^+/+^* and *Irx1^+/–^* mice at 3 dpi. Blue staining represents nuclei. E-cadherin (red) marks the epithelial layer. (**D**) Loricrin expression was examined in the *Irx1^+/+^* control gingiva. Loricrin marks the spinous and granular keratinocytes. (**E**) Loricrin expression was examined in the regenerated epithelium in *Irx1^+/+^* at 3 dpi. (**F**) Loricrin expression was examined in the *Irx1^+/–^* control gingiva. Loricrin marks the spinous and granular keratinocytes. (**G**) Loricrin expression was examined in the regenerated epithelium in *Irx1^+/–^* at 3 dpi. Arrowheads indicate Loricrin expression in the distal region of the regenerated epithelium. The regenerative front is ~600 μm to the first molar. Image area covers the region of injury. Black or white dashed lines demarcate the gingival epithelium and mesenchyme. Scale bar: 100 μm. RF, regenerative front; T, tooth.

Under homeostasis, epithelial stem/progenitor cells residing in the basal layer committed to the differentiation trajectory move upward and differentiate into KCs in suprabasal layers. As KCs undergo terminal differentiation, they become corneocytes, a group of anucleate cells that eventually form the stratum corneum layer (Figure 5A) (2, 34, 35). Although the exact KC activities during wound healing in the gingiva are still largely unknown, several studies have demonstrated how KC activities are regulated during cutaneous wound healing. The differentiation process during reepithelization of basal cells transitioning into suprabasal cells (spinous and granular KCs) occurs in concert with migration, in a wound time–specific and proximity-specific manner (36–39). Whereas the formation of the stratum corneum is suspended in the wound-activated KCs, it is restored when reepithelization is complete after injury (34, 38–40). By performing H&E staining, we observed at 3 dpi that the stratum corneum layer was formed in the RE of *Irx1^+/+^* mice, including the region of the RF (Figure 5B). In the RF and the wound periphery of *Irx1^+/–^* mice, however, the stratum corneum layer was not formed, as evidenced by the presence of nucleated cells on the surface of the RE (Figure 5C). The lack of stratum corneum indicates an incomplete KC terminal differentiation in the *Irx1^+/–^* mouse RE. To evaluate the terminal differentiation in both genotypes, we costained for the epithelial marker E-cadherin and terminal differentiation marker Loricrin at 3 dpi (Figure 5, D–G). In uninjured control *Irx1^+/+^* mouse gingiva, E-cadherin was expressed mainly in the basal and spinous layer, and Loricrin marked the differentiated cell types in the suprabasal layer (Figure 5D). In the RE, E-cadherin extended its expression in all cells of the RF. Although the thickness of the RE was not recovered to homeostatic level, Loricrin expression was already observed in the differentiated KC types from the wound periphery through the RF in *Irx1^+/+^* mice (Figure 5E). In the homeostatic epithelium of *Irx1^+/–^* uninjured mice, a similar expression level and pattern of E-cadherin and Loricrin was observed compared with *Irx1^+/+^* WT mice (Figure 5F). Nevertheless, the RF in *Irx1^+/–^* mice did not express Loricrin, as its expression can only be seen in the distal part of the wound periphery (Figure 5G). These data indicate that KC terminal differentiation was defective at 3 dpi in RE of *Irx1^+/–^* mice.

**Figure 6:**
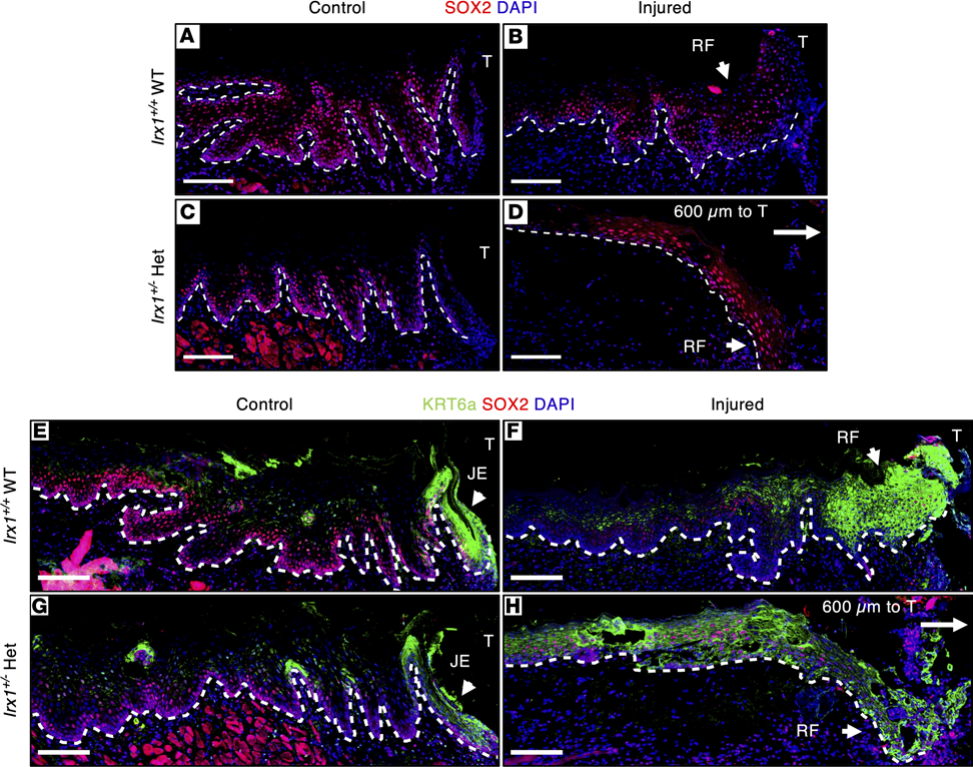
The regenerated epithelium of *Irx1^+/–^* mice showed prolonged high SOX2 and KRT6 expression at 3 dpi. (**A**–**D**) Representative IF staining images of SOX2 in control and injured gingival tissue of *Irx1^+/+^* and *Irx1^+/–^* mice at 3 dpi. (**A**) SOX2 expression was assessed in the *Irx1^+/+^* control gingiva. SOX2 marked the basal and spinous layer. (**B**) SOX2 expression was assessed in the regenerated epithelium in *Irx1^+/+^* at 3 dpi. (**C**) SOX2 expression was assessed in the *Irx1^+/–^* control gingiva. SOX2 marked the basal and spinous layer. (**D**) SOX2 expression was assessed in the regenerated epithelium in *Irx1^+/–^* at 3 dpi. The regenerative front is ~600 μm to the first molar. (**E**–**H**) Representative IF staining images of SOX2 and Keratin 6A in control and injured gingival tissue of *Irx1^+/+^* and *Irx1^+/–^* mice at 3 dpi. (**E**) KRT6A expression was assessed in the *Irx1^+/+^* control gingiva and costained with SOX2. KRT6A marks the JE and spinous and granular keratinocytes. (**F**) KRT6A expression was assessed in the regenerated epithelium in *Irx1^+/+^* at 3 dpi and costained with SOX2. (**G**) KRT6A expression was assessed in the *Irx1^+/–^* control gingiva and costained with SOX2. KRT6A marks the JE and spinous and granular keratinocytes. (**H**) KRT6A expression was assessed in the regenerated epithelium in *Irx1^+/–^* at 3 dpi and costained with SOX2. The regenerative front is ~600 μm to the first molar. Blue staining represents nuclei. White dashed lines demarcate the gingival epithelium and mesenchyme. Image area covers the region of injury. Scale bar: 100 μm. RF, regenerative front; T, tooth; JE, junctional epithelium.

We next wanted to determine the mechanism of defective KC differentiation in *Irx1^+/–^* mice . The oral mucosa is primed with genes that are not normally expressed in the epidermis, including *Sox2* and *Pitx1* (7). Epidermal overexpression (OE) of *Sox2* results in a less differentiated but more wound-activated phenotype, which accelerates cutaneous wound healing by activating proliferation and migration and by suppressing differentiation (8). We performed IF staining of SOX2 to assess its expression in the RE at 3 dpi (Figure 6, A–D). SOX2 protein was expressed in the basal and spinous layers in uninjured *Irx1^+/+^* mice (Figure 6A). At 3 dpi, the expression of SOX2 in the wound periphery was similar to uninjured controls and the layers of the RF expressed SOX2 (Figure 6B). The uninjured *Irx1^+/–^* mouse oral epithelia showed decreased SOX2 expression especially near the JE compared with *Irx1^+/+^*epithelia (Figure 6C). Interestingly, the *Irx1^+/–^* Het injured mice demonstrated ectopic expression of SOX2 in all layers of the RE (Figure 6D). Keratin 6A (*Krt6a*) is a direct downstream target of SOX2, which activates the *Krt6a* promoter in oral epithelial cells (41). KRT6A is a wound-activated keratin that is extensively upregulated in the RE during reepithelization upon wounding and returns to normal levels in the wound periphery when reepithelization is complete (42). In uninjured control epithelia, KRT6A is expressed in the spinous and granular layers and in the JE in *Irx1^+/+^* mice (Figure 6E). However, the expression of KRT6A is decreased in these layers as well as the JE of the *Irx1^+/–^* mice (Figure 6G). Interestingly, KRT6A expression was only observed in the RF of injured *Irx1^+/+^* mice; however, it was found in all layers of the injured *Irx1^+/–^* mice RE, even in the wound periphery at 3 dpi (Figure 6, F and H). This ectopic expression pattern of SOX2 and KRT6A can still be observed in the 7 dpi *Irx1^+/–^* mice. KRT6A is a hallmark of psoriasis, a disease resulting from ectopic proliferation and dysregulated differentiation of KCs (42–44). The epidermal differentiation markers, including Loricrin, are reduced in psoriatic lesions, which also exhibit nucleated corneocytes (44). The prolonged KRT6A expression and reduced Loricrin expression in the RE of injured *Irx1^+/–^* mice demonstrate a psoriatic phenotype of gingival wound healing in these mice. Taken together, these data suggest defective and delayed KC differentiation in the RE of injured *Irx1^+/–^* mice, which may result from the prolonged SOX2 and KRT6A expression in the wound periphery.

### Impaired KC migration in Irx1^+/–^ HET mice.

Two different models have been proposed during cutaneous wound reepithelization for KC migration: (a) migrating Keratin 5^+^ (KRT5^+^) basal KCs move into the wound bed and transform into suprabasal KCs in a unidirectional manner, and (b) epidermal cells in the wound periphery migrate into the wound bed by crawling or leaping over one another and then dedifferentiate to form basal cells (45, 46). The former model was supported by lineage tracing the activity of suprabasal KC, showing that dedifferentiation of suprabasal to basal KCs did not occur during cutaneous wound healing (36, 37, 47).

**Figure 7:**
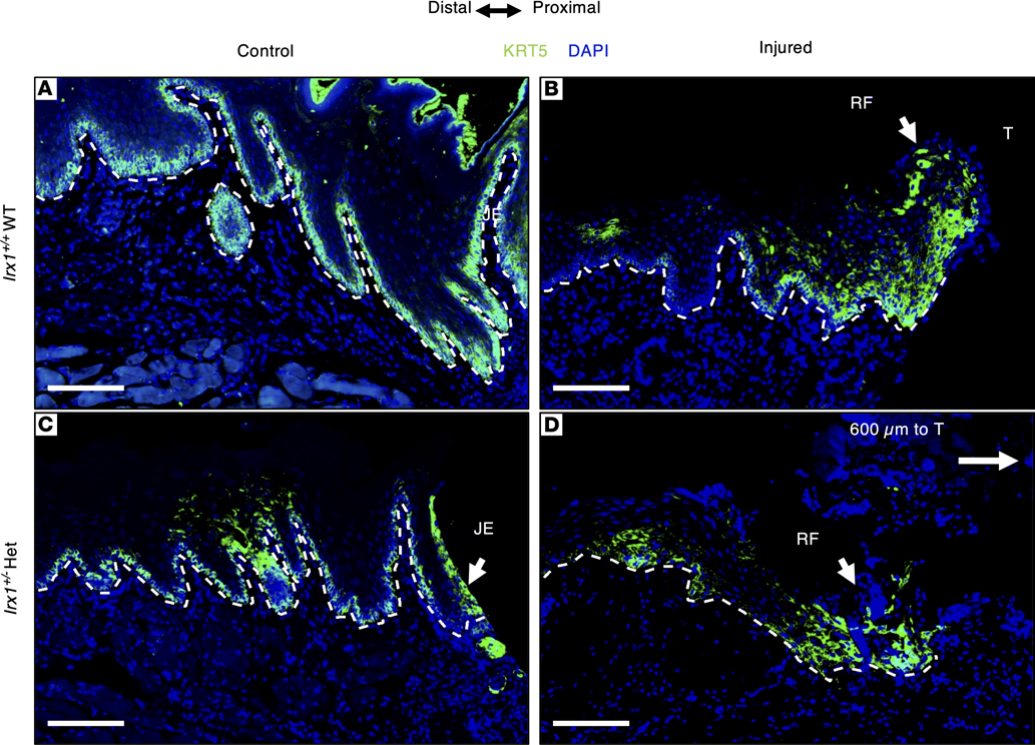
*Irx1^+/–^* mice have reduced regenerated epithelium and less KRT5^+^ cells in the RF at 3 dpi. (**A**) Representative IF staining of *Irx1^+/+^* homeostatic gingiva in front of the first molar. KRT5 marks the basal layer of gingival epithelium as well as the junctional epithelium. (**B**) Representative IF staining of the regenerated epithelium in *Irx1^+/+^*. KRT5 marks the cells in the basal layer at the distal area and the cells in the regenerative front. (**C**) Representative IF staining of *Irx1^+/–^* homeostatic gingival in front of the first molar. KRT5 marks the basal layer of gingival epithelium as well as the junctional epithelium. (**D**) Representative IF staining of the regenerated epithelium in *Irx1^+/–^*. KRT5 marks the cells in the basal layer at the distal area and the cells in the regenerative front. The regenerative front is ~600 μm to the first molar. Image area covers the region of injury. Blue staining represents nuclei. White dashed lines demarcate the gingival epithelium and mesenchyme. Scale bar: 100 μm.

To examine if KC migration was affected during reepithelization in *Irx1^+/–^* mice, we first stained for *KRT5*, which labels the basal KCs in uninjured *Irx1^+/+^* WT mouse GE and JE tissues (Figure 7A). At 3 dpi, KRT5 was expressed in the RF, as well as the basal layers at the wound periphery of injured *Irx1^+/+^* mice (Figure 7B). Homeostatic GE and JE showed reduced KRT5 expression in *Irx1^+/–^* mice (Figure 7C). At 3 dpi, we observed reduced basal cell expression of KRT5 (Figure 7D). In addition, there were fewer KRT5^+^ cells in the RF, which may suggest an altered KC proliferation and migration during gingival wound healing in *Irx1^+/–^* mice. KRT5 marks the cells in the RF upon wound healing, which is necessary for KC proliferation and migration (48, 49).

We determined the effect of IRX1 on cell proliferation in LS-8 oral epithelial cells that stably overexpress Irx1. In these cells, CCND2 expression was not increased; however, proliferating cell nuclear antigen (PCNA) expression was increased over 2-fold compared with LS-8 cells without IRX1 OE (Supplemental Figure 6A). PCNA recruits factors for DNA replication, repair, and chromatin remodeling and is associated with cell proliferation (50). OE of IRX1 in LS-8 cells was confirmed by Western blots (Supplemental Figure 6B) and resulted in increased cell proliferation (Supplemental Figure 6C). We identified decreased expression of proliferation markers CCNA2 and MKI-67 in *Irx1^+/–^* 3 dpi RE tissues; however, CCND2 expression was increased (Supplemental Figure 6D). These data suggest that IRX1 indirectly regulates cell proliferation through the activation of other transcription factors.

**Figure 8:**
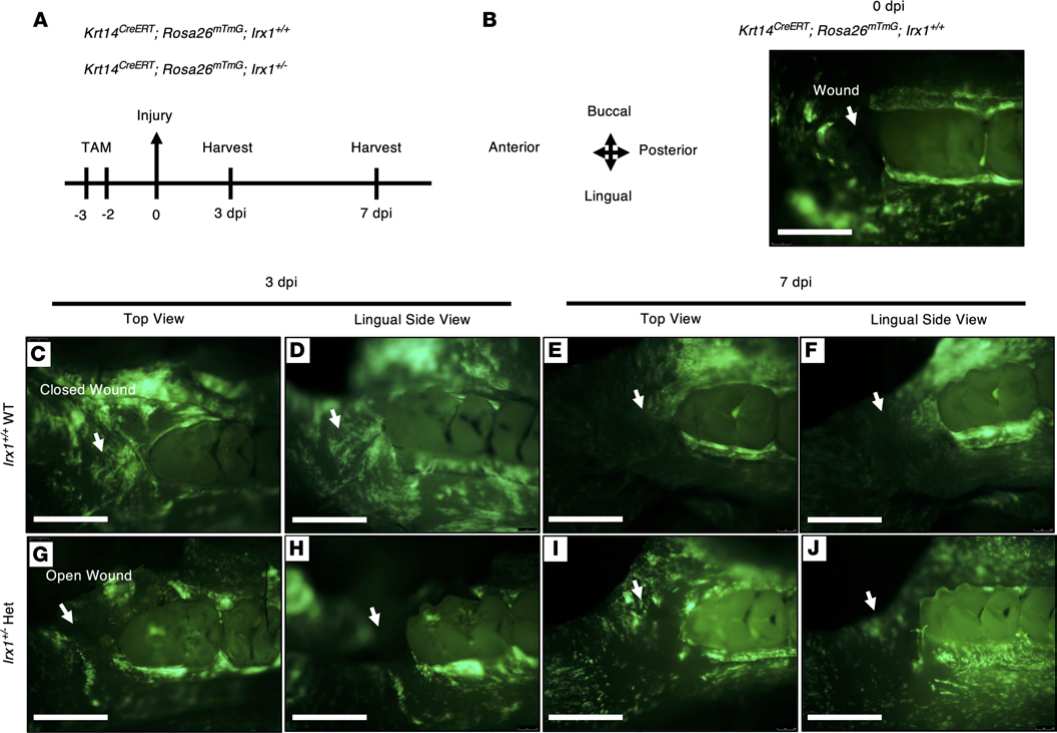
Lineage tracing of KRT14*^+^* basal cells upon wounding in the oral epithelia of *Irx1^+/+^* and *Irx1^+/–^* mice. (**A**) Timeline of the lineage tracing experiments for both *Krt14^CreERT^;Rosa26^mTmG^;Irx1^+/+^* and *Krt14^CreERT^;Rosa26^mTmG^;Irx1^+/–^* groups. Mice from both groups were induced with Tamoxifen for 2 consecutive days at 12–16 weeks old and injured 2 days after the second dose. Mandibular tissues were harvested at 0, 3, and 7 dpi for imaging and further analysis. (**B**) A representative image showing the successful induction of Cre expression (GFP signal) and tissue removal in front of the first molar. (**C** and **D**) Representative images of the basal cell activity at 3 dpi in *Irx1^+/+^*. (**E** and **F**) Representative images of the basal cell activity at 7 dpi in *Irx1^+/+^*. (**G** and **H**) Representative images of the basal cell activity at 3 dpi in *Irx1^+/–^*. (**I** and **J**) Representative images of the basal cell activity at 7 dpi in *Irx1^+/–^*. Scale bar: 1 mm. Arrows point to the wound site.

To trace the basal cell activity upon wounding in the oral epithelium, we performed lineage tracing using *Krt14^CreERT^;Rosa26^mTmG^;Irx1^+/+^* and *Krt14^CreERT^;Rosa26^mTmG^;Irx1^+/–^* mice (Figure 8). We first determined if KRT14 expression was affected in the *Irx1^+/–^* mice. We stained for KRT14 in *Irx1^+/+^* and *Irx1^+/–^* mice and found no change in KRT14 expression in the *Irx1^+/–^* mice compared with *Irx1^+/+^* mice (Supplemental Figure 7). Mice were injected with Tamoxifen for 2 consecutive days, and gingival injury was performed 2 days after the last dose. Tissues were then collected at 0, 3, and 7 dpi for analysis (Figure 8A). The oral epithelia basal cell layer expressing *Krt14^Cre^* was induced with Tamoxifen causing the expression of GFP from the *Rosa26^mTmG^* reporter. At 0 dpi (after the surgery), the GFP signal was mostly concentrated in the gingiva around the molar row. As a control, we show that the gingiva in front of the first molar was successfully removed after the surgery (Figure 8B). At 3 dpi, increased GFP signal at the injury site indicated the reepithelization process was near completion in *Irx1^+/+^* WT mice (Figure 8, C and D). At 7 dpi, the GFP signal was diluted in the wound bed, indicating the turnover of the RE in *Irx1^+/+^* mice (Figure 8, E and F). In *Irx1^+/–^* mice at 3 dpi, the GFP signal was absent at the wound area, and the GFP pattern was still mainly concentrated in the molar row, which was largely similar to 0 dpi (Figure 8, G and H). At 7 dpi, the GFP signal was expanded and could be seen at the wound bed; thus, GFP retaining cells migrated to close the wound, indicating oral tissue regeneration (Figure 8, I and J). These data demonstrate that *Irx1* regulates basal cell proliferation and stratification of the layers of the gingiva during wound healing.

**Figure 9:**
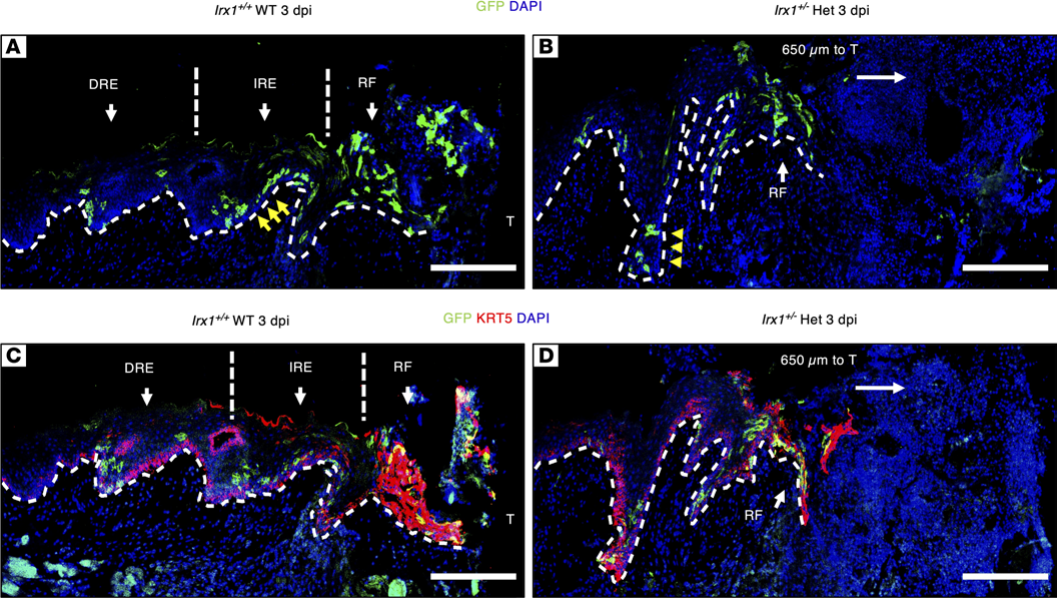
Delayed KC proliferation and migration in *Irx1^+/–^* mice at 3 dpi. (**A**) Representative IF image staining for GFP in *Irx1^+/+^* WT 3 dpi mice. Yellow arrows indicate the expanded basal/spinous population. (**B**) Representative IF image staining for GFP in *Irx1^+/–^* Het 3 dpi mice. Yellow arrowheads indicate the smaller basal/spinous population. The RF is 650 μm to the tooth. (**C**) Representative IF image for GFP and KRT5 costaining in *Irx1^+/+^* mice. (**D**) Representative IF image for GFP and KRT5 costaining in *Irx1^+/–^* mice. The RF is 650 μm to the tooth. Blue staining represents nuclei. RF, regenerative front; IRE, intermediate regenerated epithelium; DRE, distal regenerated epithelium; T, tooth. White dashed lines demarcate the gingival epithelium and mesenchyme. Scale bar: 200 μm.

The IF signal of GFP to assess the basal cell activities during gingival wound healing showed that cells at the wound edge migrated toward the tooth to form the RE. During healing, the RE can be separated into 3 zones according to the previous research: the RF that is highly migrative, intermediate RE (IRE) that is more proliferative than migrative, and distal RE (DRE) that has a return-to-normal gene expression signature (36, 37, 47). At 3 dpi, the basal layer and spinous layer cells were labeled with GFP in the RF of *Irx1^+/+^* mice (Figure 9A). In the IRE, there are several GFP^+^ cell clusters in the basal/spinous layers. There were decreased cell clusters observed in the DRE zone (Figure 9A). Furthermore, in *Irx1^+/+^* mice at 3 dpi, the KRT5^+^ cells, which represented the division of the basal cells and progenitor cell production to provide a cell source for reepithelization, were observed in the basal and spinous layers and RF (Figure 9C). In the RF of *Irx1^+/–^* mice at 3 dpi, there were less KRT5^+^ cells, indicating a smaller, less active RF structure compared with *Irx1^+/+^* mice (Figure 9D). We could not distinguish the IRE and DRE in the *Irx1^+/–^* mice at this stage, demonstrating defective regenerated epithelia. Moreover, we did not observe GFP^+^ cells in the upper suprabasal layer in the RE of *Irx1^+/–^* mice, indicating a defect in basal cell stratification, consistent with previous results (Figure 9B). In summary, at 3 dpi, *Irx1^+/–^* mice exhibited a smaller migrative zone and less proliferative cell clusters, revealing a defect in proliferation of basal cells and stratification of differentiating cells during reepithelization.

### RNA-Seq reveals a wound-activated transcriptome in Irx1^+/+^ mice, which is diminished in Irx1^+/–^ mice upon injury.

**Figure 10:**
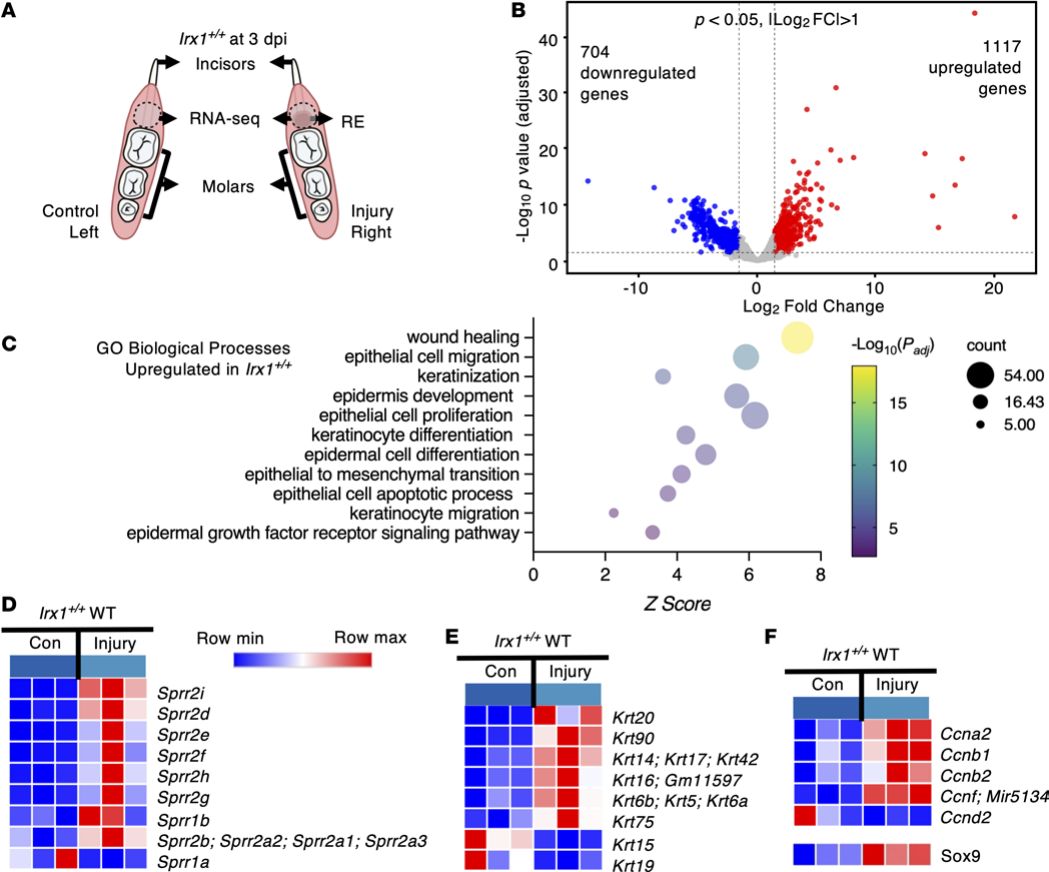
Upregulation of the wound-activated transcriptome in the RE of *Irx1^+/+^* mice at 3 dpi. (**A**) Schematics showing the left (control) and right (injured) mandibles of *Irx1^+/+^* mice and the tissue analyzed by RNA-Seq. *n* = 3. (**B**) Volcano plot showing differentially expressed genes between the control and injured gingiva at 3 dpi in *Irx1^+/+^* mice. (**C**) GO analysis of the 1,117 significantly upregulated genes in the injured tissue over the control. *P*_adj_ < 0.05. (**D**) The heatmap showing the relative mRNA expression levels of small proline-rich protein (SPRR) family members in the control and the injured gingiva in *Irx1^+/+^* mice. (**E**) Heatmap showing the relative mRNA expression levels of keratins in the control and the injured gingiva in *Irx1^+/+^* mice. (**F**) The heatmap showing the relative mRNA expression levels of genes related to proliferation in the control and the injured gingiva in *Irx1^+/+^* mice. RE, regenerated epithelium.

To understand the molecular mechanisms of regeneration and reepithelization of oral gingival epithelia, we performed total RNA-Seq of *Irx1^+/+^* mice at 3 dpi. We first assessed the RNA expression profile between *Irx1^+/+^* injured and uninjured oral epithelia (Figure 10A). There were 1,117 upregulated genes and 704 downregulated genes in the injured gingiva compared with uninjured (*P* < 0.05; |log_2_ FC| > 1) (Figure 10B). By performing gene ontology (GO) analysis on the 1,117 upregulated genes, we identified several significantly regulated GO terms that related to epithelial wound healing (Figure 10C). Several small proline-rich proteins (SPRR), which belong to the epidermal differentiation complex (EDC) (51), were significantly upregulated in the injured tissue over the control in *Irx1^+/+^* mice, except *Sprr1a*, which was downregulated (Figure 10D). We also assessed keratin mRNA levels from both groups. Basal cell–specific KRT5 and KRT14 as well as wound-activated KRT6, KRT16, and KRT17 were significantly upregulated upon wounding in the *Irx1^+/+^* mice compared with controls. *Krt15*, which has been reported to be suppressed in the wound-activated skin epithelium, was downregulated in the injured *Irx1^+/+^* group (Figure 10E) (52). Cyclin genes were also identified as being differentially expressed, with most of them found to be upregulated in the injured tissue, with the exception of Ccnd2 (Figure 10F). Interestingly, the *Sox9* gene, a known transcription factor that regulates stem/progenitor cells in the skin, was also upregulated in the injured gingiva tissue in the *Irx1^+/+^* mice at 3 dpi (53, 54). Previously, we reported that SOX9 expression was decreased in the *Irx1^–/–^* null embryo (10). This mRNA expression profile revealed a wound-activated gene expression feature in the injured tissue of *Irx1^+/+^* mice compared with the uninjured control at 3 dpi. The regenerated tissue exhibited enhanced proliferation, migration, and differentiation at 3 dpi.

**Figure 11:**
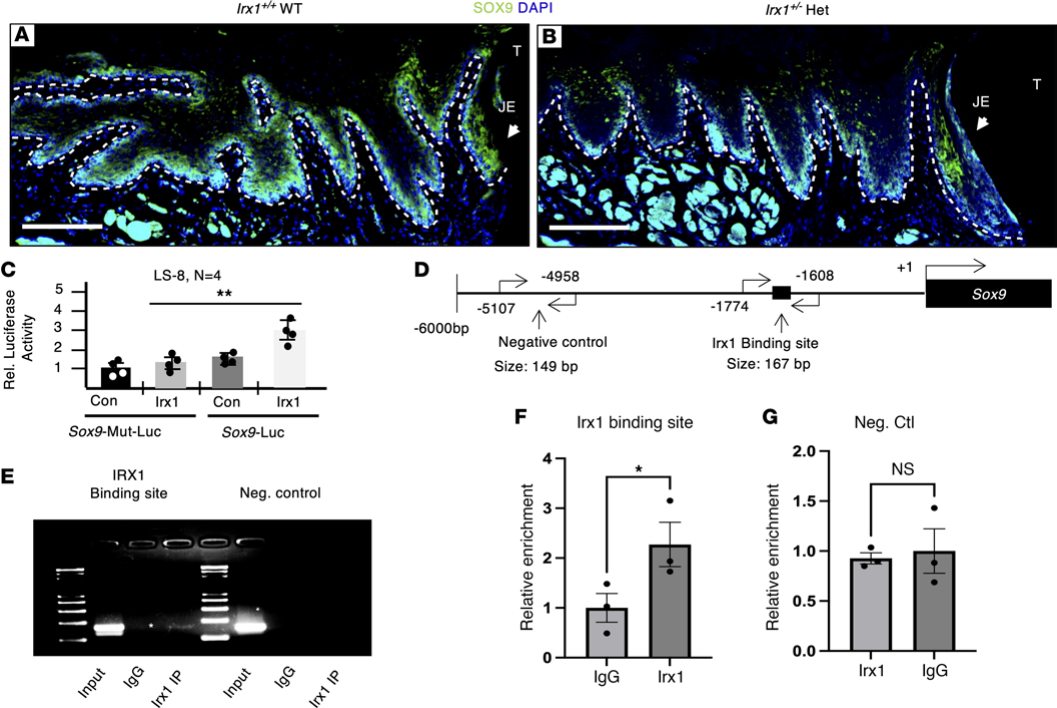
IRX1 regulates SOX9 expression. (**A**) Representative IF staining image showing the SOX9 expression in the homeostatic gingiva of *Irx1^+/+^* mice. (**B**) Representative IF staining image showing the SOX9 expression in the homeostatic gingiva of *Irx1^+/–^* mice. (**C**) *Irx1* plasmid or a control empty vector were overexpressed along with the WT *Sox9* promoter fused to luciferase reporter or with a promoter containing a mutated canonical Irx1 binding site. *n* = 4. (**D**) A schematic of the *Sox9* promoter, highlighting the IRX1 binding site and the negative control regions that were amplified by the given primer sets in the ChIP assay. (**E**) Gel-electrophoresis of PCR amplified products from the input and ChIP reactions using primers flanking the IRX1 binding site and the negative control region. The specific band amplified is labeled with an asterisk. (**F**) qPCR demonstrating an enrichment using primers flanking the IRX1 binding site when the specific antibody was used compared with the nonspecific control. *n* = 3. (**G**) There is no difference in the samples pulled down with IgG and the specific IRX1 antibody when the primer set flanking a control region lacking an IRX1 binding site is used. *n* = 3.

In support of IRX1 regulation of SOX9 expression, we performed IF staining for SOX9 in *Irx1^+/+^*and *Irx1^+/–^*mouse oral epithelium. SOX9 is expressed in the basal cell layer in *Irx1^+/+^* mice and decreased in *Irx1^+/–^* mice (Figure 11, A and B). To demonstrate direct IRX1 regulation of the *Sox9* promoter, the *Sox9* promoter containing the IRX1 binding site (5’*ACAnnTGT*3’) was cloned into a luciferase plasmid. As a control, the *Irx1* sequence was mutated in the *Sox9* promoter, and both the WT sequence and mutant sequence were tested for luciferase activity with and without IRX1 OE in LS-8 oral epithelial cells (Figure 11C). IRX1 activated the *Sox9* promoter but not the *Sox9* promoter with a mutated IRX1 binding site (Figure 11C). We next used ChIP experiments in OCCM30 cells, which express IRX1 and SOX9, to determine in vivo binding of IRX1 to the *Sox9* promoter (Figure 11D). Primers flanking a negative control region and the IRX1 binding site were designed and used to amplify input chromatin, and used to amplify chromatin resulting from ChIP reactions using nonspecific IgG and a monoclonal IRX1 antibody. Both sets of primers amplified a product from input chromatin samples (Figure 11E), and the primers flanking the IRX1 binding site only amplified a product when the specific antibody was used to perform the ChIP. The negative control primers did not amplify a product when either antibody was used. Using qPCR, the enrichment of the chromatin containing the IRX1 binding site precipitated with the specific antibody was confirmed (Figure 11F). There was no enrichment with the negative control primer set using either antibody to precipitate chromatin (Figure 11G).

**Figure 12:**
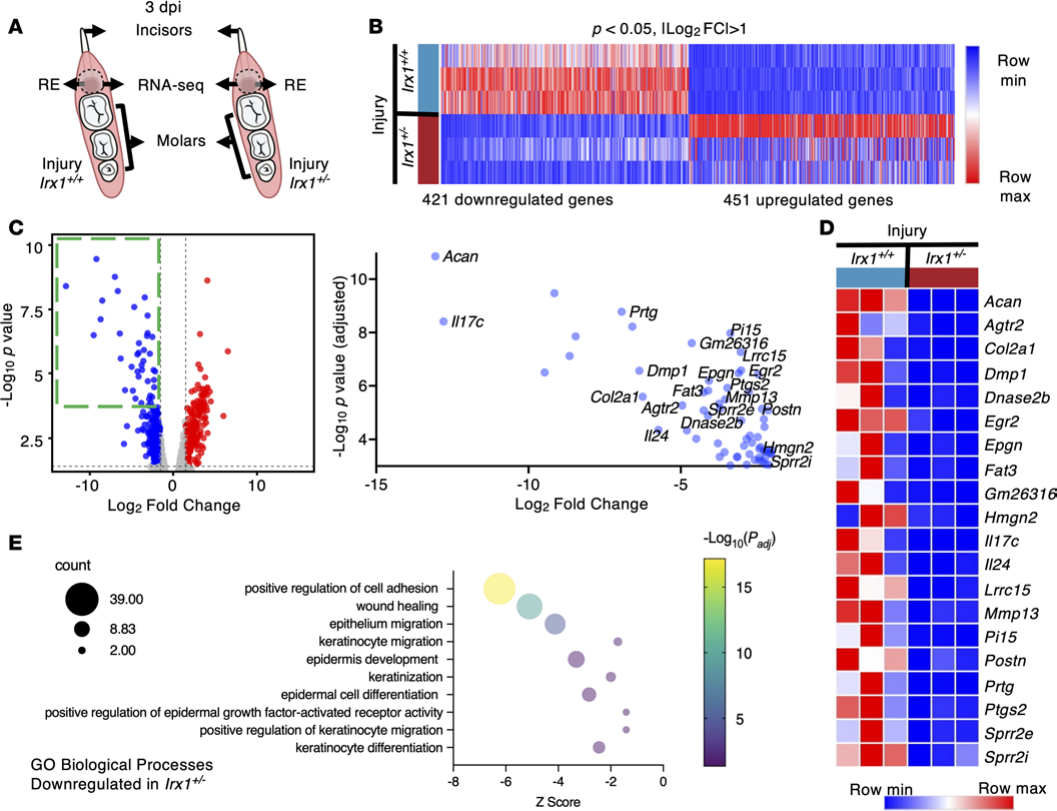
RNA-Seq analysis showed downregulation of wound healing–related genes in the injured gingiva of *Irx1^+/–^* Het mice. (**A**) Schematics showing the *Irx1^+/+^* WT and *Irx1^+/–^* Het injured mandibles at 3 dpi and the tissue collected for RNA-Seq. *n* = 3. (**B**) Heatmap showing the significantly up- and downregulated genes in the injured *Irx1^+/–^* Het mouse tissue compared with *Irx1^+/+^* WT mouse injured tissue. (**C**) Volcano plot displaying differentially expressed genes between the control and injured gingiva at 3 dpi in *Irx1^+/+^* WT mice. The right panel showed the hits with –log_10_ P > 3 and Log_2_ FC < –2. Some of the most significantly downregulated genes in the *Irx1^+/–^* mice were highlighted. (**D**) The heatmap showing the most downregulated genes in the *Irx1^+/–^* Het mice. (**E**) GO analysis of the 421 significantly downregulated genes in the injured tissue of *Irx1^+/–^* Het mice over the *Irx1^+/+^* WT mice. *P_adj_* < 0.05. RE, regenerated epithelium.

To determine the molecular mechanisms of defective reepithelization in *Irx1^+/–^* mice at 3 dpi, we observed the mRNA expression profile of the RE in *Irx1^+/+^* mice compared with *Irx1^+/–^* mice (Figure 12A). The analysis identified 451 upregulated genes and 421 downregulated genes significantly differentially expressed in the *Irx1^+/–^* injured tissues (*P* < 0.05; |log_2_ FC| > 1) (Figure 12, B and C). We then analyzed the genes with more strict parameters (-Log_10_ *P* > 3 and log_2_ FC < –2), and identified the top genes that were downregulated in *Irx1^+/–^* Het injured tissue compared with control injured tissue (Figure 12, C and D). Genes upregulated upon wound healing in the epithelial tissue or positively regulating wound healing were significantly downregulated in the injured *Irx1^+/–^* Het mice. Several genes, including *Epgn*, a ligand of EGF signaling pathway regulating KC migration and proliferation (5, 6, 55, 56); *Mmp13*, a matrix metalloproteinase (MMP) that regulates KC migration; and *Postn*, an essential gene for KC proliferation during cutaneous wound healing (57), were all decreased in *Irx1^+/–^* injured mice (Figure 12D). SPRR2E and SPRR2I regulate KC differentiation (51), DNASE2B controls DNA degradation during KC cornification and regulates their terminal differentiation (58), and IL-17C and IL-24 — encoding 2 epithelial ILs, which are highly upregulated during cutaneous wound healing in the KCs and regulate their migration and proliferation (59–61) — are all decreased in *Irx1^+/–^* injured mice (Figure 12D). PTGS2 is upregulated during skin wound healing (62, 63) and is decreased in *Irx1^+/–^* injured mice (Figure 12D).

GO analysis of the 421 downregulated genes revealed significant GO terms related to wound healing and revealed positive regulation of KC migration and KC differentiation (Figure 12E). The RNA-Seq data identify genes and pathways that are regulated by IRX1 and that are positively related to wound healing, regeneration, and reepithelization. These data demonstrate the role of IRX1 in positively regulating the wound healing process.

### EPGN expression is activated during oral epithelial regeneration while its expression is decreased and delayed in Irx1^+/–^ HET injured mice.

**Figure 13:**
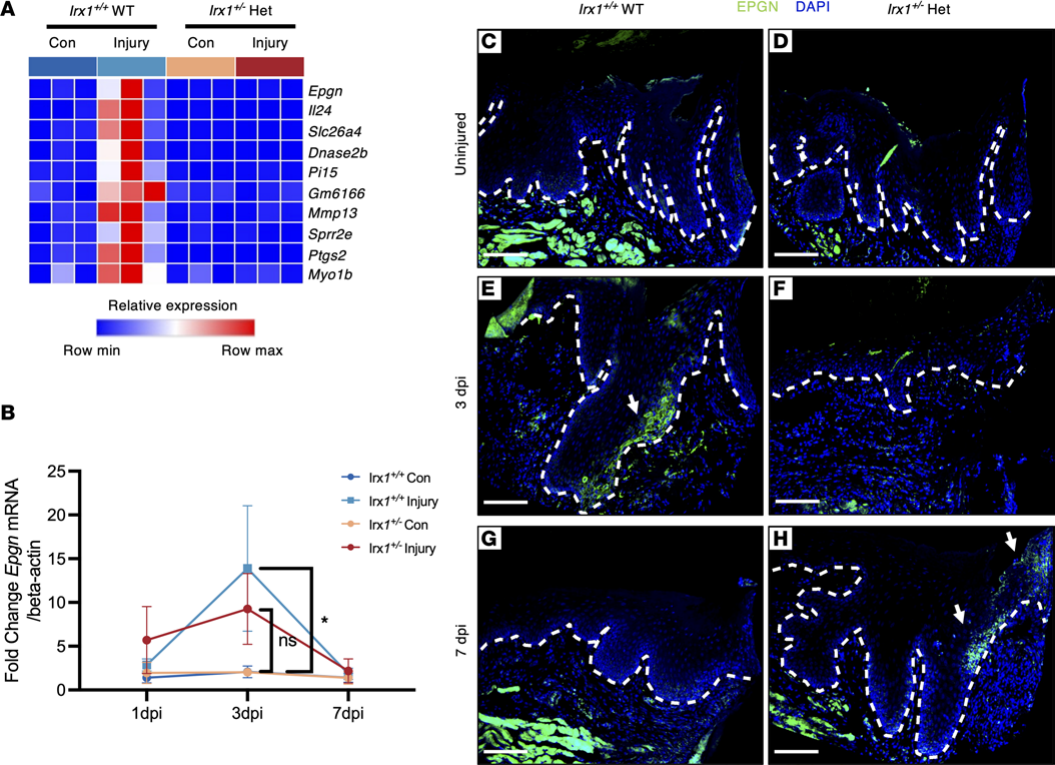
Decreased EPGN underlies the delayed reepithelization in *Irx1^+/–^* mice. (**A**) Heatmap showing the relative mRNA expression levels of a subset of the significant genes upregulated in the injured tissue of *Irx1^+/+^* mice over uninjured *Irx1^+/+^* mice as well as the injured tissue of *Irx1^+/–^* mice. (**B**) qPCR detected the mRNA level of *Epgn* at 1, 3, and 7 dpi. *n* = 3 (**C** and **D**) Representative IF staining of EPGN protein in the unwounded gingiva of *Irx1^+/+^* and *Irx1^+/–^* mice. (**E** and **F**) Representative IF staining of EPGN protein in the injured gingiva of *Irx1^+/+^* and *Irx1^+/–^* mice at 3 dpi. (**G** and **H**) Representative IF staining of EPGN protein in the injured gingiva of *Irx1^+/+^* and *Irx1^+/–^* mice at 7 dpi. Blue staining represents nuclei. Scale bar: 100 μm.

We identified 10 genes whose activation were impaired due to *Irx1* haploinsufficiency upon gingival wounding (Figure 13A). Among these genes, epithelial mitogen (*Epgn*) has been identified as one of the most significantly differentially expressed and was induced only in the *Irx1^+/+^*mouse injured tissue. It is a ligand of the EGF signaling pathway, which is essential to control wound healing by regulating KC migration, proliferation, differentiation, and epidermal-mesenchymal transition (5, 6, 55, 56). EPGN is induced upon injury in the cutaneous wound (47, 64). We performed qPCR of *Epgn* mRNA levels in 1, 3, and 7 dpi samples from both genotypes, confirming that the induction of *Epgn* was reduced in the *Irx1^+/–^* injured tissue (Figure 13B). EPGN protein was not expressed in the homeostatic (uninjured) GE or JE in either genotype (Figure 13, C and D). At 3 dpi, EPGN was activated in the leading edge of the RE in the *Irx1^+/+^* mice but was not observed in the RE of *Irx1^+/–^* mice (Figure 13, E and F). At 7 dpi, EPGN expression returned to normal levels in the RE of *Irx1^+/+^* mice, while some induction of EPGN was observed in the RF of *Irx1^+/–^* mice (Figure 13, G and H). The delayed onset of EPGN expression upon wounding in *Irx1^+/–^* mice suggests a role of EPGN in mediating *Irx1* regulation of oral epithelial regeneration and reepithelization.

To demonstrate a direct role for EPGN in cell migration, we performed a scratch assay to determine if the addition of EPGN to LS-8 oral epithelial cells would increase cell migration and scratch closure. Oral epithelial cells (LS-8) were transfected with either *Irx1*, *sh-control* (con), or *sh-Irx1* (inhibits IRX1 expression) plasmids (1 μg) and a uniform scratch was made to remove cells from the plate area. Scratch closure was monitored at 0 hours (T0), T12, and T24. Inhibition of endogenous IRX1 (*sh-Irx1*; Supplemental Figure 6B) reduced cell migration compared with LS-8, LS-8 OE IRX1, and LS-8 *sh-con* cells (Supplemental Figure 8A). However, the addition of EPGN (0.5 ng/μL) to cell cultures prior to scratch assay rescued cell migration when IRX1 expression was inhibited by *sh-Irx1* (Supplemental Figure 8B). Quantitation of the scratch assays are shown in (Supplemental Figure 8C).

In addition, cell proliferation was increased with IRX1 OE and decreased with *sh-Irx1* (inhibition) (Supplemental Figure 9A). Addition of EPGN to cell cultures rescued cell proliferation after 72 hours (T72), when IRX1 expression was inhibited by *sh-Irx1* (Supplemental Figure 9B). We have shown that IRX1 regulates both SOX9 and EPGN expression; we asked if EPGN activated *Sox9* and *Irx1* transcripts. Interestingly, EPGN treatments activated both SOX9 and IRX1 expression, suggesting a potential feedback mechanism for the regulation of these pathways (Supplemental Figure 9C).

## Discussion

The increasing prevalence of chronic wounding and scarring, which affects people’s quality of life and increases healthcare costs, has become more prominent (3). The gingiva, as part of the oral mucosa, has a similar structure to the skin but is primed with wound-activated gene signatures, which facilitate rapid and scarless wound healing (2, 7, 65). Thus, identifying the oral epithelial stem cell (OESC) factors that contribute to gingival wound healing can also provide insights to improve oral and dental health.

### Irx1 is primed in homeostatic gingiva and serves as a potential stem cell marker in the gingiva to activate the wound healing response.

The expression of several OESC markers, including CD44, BMI1, SOX2, KERATIN 14, and PITX1, have been described in the basal layer (7, 66). The molecular mechanisms of how these factors regulate tissue maintenance and regeneration remain largely unknown. Furthermore, the role of transient amplifying (TA) progenitor cells in the oral epithelium as a reservoir for wound healing and homeostasis is not known (66–68). TA cells derived from stem cells are more differentiated than stem cells and are highly proliferative. These cells will undergo several divisions before terminal differentiation. How stem cells contribute to the amplification of the transient cells may depend on the number of stem cells and factors within the basal layer. We are working to identify and understand the factors regulating the stem cells and TA cells in the basal layer of the oral epithelium.

This is the first report of IRX1 expression in the basal layer of GE, JE, and lamina propria at homeostasis. We initially identified IRX1 expression in oral tissues at early stages during murine development (10). Interestingly, IRX1 is expressed in discreet regions and within specific tissue niches during development. Furthermore, as development proceeds, IRX1 expression decreases and is only found in small tissue-specific niches (10). It is coexpressed with SOX2, which primes the gingiva to facilitate the fast healing of gingival wounds. The basal layer of GE and JE as well as the lamina propria are known hubs for gingiva-derived stem cells, helping to maintain tissue integrity and contribute to regeneration upon injury (25–28). The expression pattern of IRX1 makes it a potential marker for the stem cells in the gingiva oral epithelium.

### Irx1 directly regulates Sox9 expression in the OESC.

The RNA-Seq experiments also revealed increased SOX9 expression after injury in WT mice. In addition, SOX9 expression was decreased in *Irx1^+/–^* mice. We show through a series of experiments that IRX1 binds to and activates the *Sox9* promoter. SOX9 has been shown to be expressed in other OESCs, such as the luminal stem/progenitor cells and distal progenitor cells of the salivary glands (69–71). SOX9 expression contributes to the lung epithelium during development (72) and airway regeneration (73). In this report, we demonstrate a role for SOX9 expression in the oral epithelium and during wound healing. Interestingly, our data also show that SOX9 expression occurs in the suprabasal layer directly above the basal layer. These cells immediately adjacent to the basal layer may represent potential TA cells that were derived from the IRX1^+^ cells in the basal layer (74, 75). Interestingly, we show that, in the *Irx1^+/–^* Het mice, SOX9 expression is reduced in this suprabasal TA cell layer. These results demonstrate 2 factors in the oral epithelium (IRX1 and SOX9) that appear to regulate the OESC niche and a layer of suprabasal cells that may form a potential proliferative TA layer that gives rise to the differentiating cells of the spinous layer.

### Irx1 haploinsufficiency delays wound closure through altering KC differentiation and migration.

The terminal differentiation of KCs in the RE of *Irx1^+/–^* was delayed, with decreased terminal differentiation marker expression and the retaining high expression of wound-activated proteins including SOX2 and KRT6. We speculate that delayed KC differentiation was due to a larger pool of less differentiated wound-activated KCs to facilitate the wound closure or that the intrinsic KC terminal differentiation pathway was delayed by the prolonged wound-activated gene expression, even in the DRE. However, in the second scenario, the RE in *Irx1^+/–^* injured mice should exhibit a more proliferative and migrative phenotype, which was not observed in the lineage tracing experiments. Furthermore, KC differentiation has been reported to happen in concert with migration in the RE and is largely dependent on its acquired migration ability (36). CK19 is also found in human JE and restricted to the basal layer of GE (76). In localized juvenile spongiotic gingival hyperplasis, CK19 expression transitions to expression throughout the lesional epithelium. The formation of these lesions and expanded CK19 expression may be diagnostic for these gingival lesions and may be a new marker for epithelial basal cells (76).

Therefore, IRX1 modulates KC differentiation and migration through regulation of the basal stem cell layer and activation of SOX9 in proliferating TA cells (77), leading to a smaller pool of KCs to close the wound.

### Transcriptomic analysis identified a decreased wound-activated signature in the Irx1^+/–^ RE.

To determine the mechanism behind the delayed wound closure, we performed total RNA-Seq in the control and injured gingival tissue in *Irx1^+/+^* WT and *Irx1^+/–^* Het mice at 3 dpi. As a control, the *Irx1^+/+^* WT mouse RE exhibited a wound-activated phenotype with highly active KC proliferation, migration, and differentiation, while the *Irx1^+/–^* Het mouse RE showed downregulation of genes in these KC activities, indicating a diminished wound-activated signature at 3 dpi. GO terms — including KC migration, KC differentiation, wound healing, and positive regulation of EGF signaling — were negatively affected in the *Irx1^+/–^* RE compared with the *Irx1^+/+^* RE. The analysis also identified that EPGN, a ligand of the EGF signaling pathway, was not activated upon injury in the *Irx1^+/–^* Het RE at 3 dpi. IF staining of EPGN confirmed the delayed onset of EPGN expression in the RF in *Irx1^+/–^* Het mice. These data suggest that IRX1 may regulate EPGN expression in the KC in the RF and alter KC proliferation and migration through EGF signaling.

Interestingly, in injured urothelial tissues, EGFR ligands including EPGN where upregulated, and urothelial basal cells express EGFRs (78, 79). The urothelium or transitional epithelium are cells that appear to be stratified and line the inside of the kidneys, urethra, and bladder (80). The urothelium contains a basal cell layer, intermediate cells, and superficial or umbrella cells at the surface or lining of the bladder (80). The urothelial basal layer contains stem cells, which after injury are required for rapid repair and regeneration, and the renewal depends on factors expressed in both the stroma and urothelium (80). Similar to the oral epithelium, KRT14^+^ and KRT5^+^ urothelial basal cells proliferate after a urothelial injury and are the progenitors of all urothelial lineages (80). These processes are similar to repair and regeneration of the oral mucosa.

Interestingly, SOX9 is expressed in urothelium basal and intermediate cells, and EGFR induces SOX9 expression in injured urothelium (79). While we do not know if IRX1 is expressed in the urothelium, both EGFR and SOX9 contribute to urothelium repair and regeneration after injury. We speculate that IRX1 regulation of SOX9 and EPGN and potential EPGN activation of SOX9 provides a unique mechanism for the control of KC proliferation, differentiation, and migration as these 3 factors are essential for these functions in wound healing.

This research identified *Irx1* as a gene that is primed in the oral epithelia at the basal stem cell layer, and it facilitates the rapid and scarless wound healing through a IRX1/EGF/SOX9 signaling cascade. These data also provide further insights into improving treatments toward nonoral wounds.

## Methods

Supplemental Methods are available online with this article.

### Sex as a biological variant.

Our study examined male and female animals, and sex was not considered as a biological variable. The findings contain data from both male and female mice.

### Human gingiva samples.

Criteria included the selection of healthy individuals with no history of smoking or diabetes. Prior to the treatment procedure, a preprocedural mouth rinse comprising 0.2% chlorhexidine was administered. Following the infiltration of local anesthesia (2% lidocaine) in proximity to the edentulous area on the mandibular first molar, a soft tissue punch with a diameter of 4 mm was used to extract the gingival tissue at the designated surgical site. Subsequently, the excised gingival tissues were fixed in 4% paraformaldehyde for further analysis.

### Mouse strain breeding.

The *Irx1*-KO mouse strain (*Irx1^LacZNeo^*) was generated as previously described (10). The genotyping primers for Irx1 are listed below. The *Krt14^CreERT^* [strain no. 005107 STOCK Tg(*KRT14-cre/ERT*)20Efu/J] and the *Rosa26^Tomato–GFP^* [strain no. 037456, NOD.129(Cg)-Gt(ROSA)26Sortm4(ACTB-tdTomato,-EGFP)Luo/YgchJ] originated from The Jackson Laboratory. The genotyping primers for all the mouse strains are listed in Supplemental Table 1; a list of antibodies, primers for RT-PCR, and ChIP-PCR are listed in Supplemental Tables 2–5

### Statistics.

The significance cutoff for RNA-Seq analysis is |Log_2_ FC| > 1 and *q* < 0.05. Significance cutoffs for all other analyses are *P* < 0.05. All quantified results are presented as mean ± SEM, with an *n* value indicating the number of biological repeats. A 2-tailed unpaired Student’s *t* test and either 1- or 2-way ANOVA were used to determine statistical significance.

### Study approval.

Human gingival tissue samples were collected from patients scheduled for implant placement at the University of Iowa College of Dentistry, following IRB approval under protocol no. 201903760. Mice were maintained in the animal facility of the University of Iowa. All experiments were approved by the IACUC of the University of Iowa.

### Data availability.

Requests for further information should be directed to and will be fulfilled by the corresponding author. Values for all data points in graphs are reported in the Supporting Data Values file. All materials, data, and code will be available upon request and deposited in GEO repository (GSE281359).

## Author contributions

DS contributed concepualization, data curation, formal analysis, methodology, validation, and writing; TK contributed data curation, formal analysis, methodology, validation, visualization, and writing; SS contributed data curation, methodology, validation, and writing; YS contributed data curation, formal analysis, methodology, visulaization, and writing; MS contributed data curation, formal analysis, methodology, validation, and writing; FS contributed data curation, methodology, validation, and writing; SE contributed data curation, formal analysis, methodology, project administration, validation, and writing; BAA contributed conceptualization, formal analysis, funding acquisition, investigation, project administration, resources, supervision, and writing.

## Supplementary Material

Supplemental data

Unedited blot and gel images

Supporting data values

## Version 1 01/09/2025

Electronic publication
