# Supplementary material for: Irx1 mechanisms for oral epithelial basal stem cell plasticity during reepithelialization after injury
Source: JCI Insight. 2025 Jan 9;10(1):e179815. doi: 10.1172/jci.insight.179815 (PMC11721312; doi:10.1172/jci.insight.179815)
Supplement: Supplemental data [file jciinsight-10-179815-s233.pdf]

SUPPLEMENTAL DATA

Methods

Mouse gingival injury model

Male and female adult mice (10-16 weeks old) were anesthetized with 2-5% isoflurane in an induction chamber connected to an evaporator. The mice were then switched to the nose cone and onto a surgical platform with a heated pad. The mouth was opened with a self-retaining retractor (WPI) to ensure visual access to the lower molar rows. Puncture wounds were made using a disposable biopsy punch (0.75mm, Robbins Instruments) anterior of the first molar on the right side of the mandible to remove the gingiva (gingival epithelium and junctional epithelium included). Mice were injected with 2 mg/kg Buprenorphine (ZooPharm) for analgesia right after wounding. Mice were returned to the facility upon recovery from anesthetization and fed with a regular diet (hard pellet) daily. Animals were then harvested on 0, 1, 3 and 7 day-post-injury (dpi) to determine the wound healing process. All instruments were autoclaved every time before each surgery.

For genetic lineage-tracing experiments of $Krt14^{CreERT}; Rosa26^{mTmG}$ mice, male and female adults were given one dose of tamoxifen intraperitoneally per day for two consecutive days (40 mg/kg body weight per dose). Mice were wounded 48 h after the second dose of tamoxifen injection and harvested on 0, 3, 7 and 14 dpi. The mandibles were dissected out and observed using a Leica M165 FC fluorescence stereomicroscope to access GFP expression. Tissues were then processed for H&E and GFP staining.

The operator was blinded to the genotype, age, and day post injury for sample collections.

Quantitation of wound size

0 days, 1 day and 3 days after wounding, mice were euthanized and the mandible tissue was dissected out. Images of the wound site were taken with different magnifications using a dissection scope. The wound area was selected with the polygon tool and measured in FIJI ImageJ 3 times. An average area of the three values was calculated to represent the area of the wound.

Total RNA Sequencing

Injured (right side) and uninjured (left side) gingival tissues over the wound bed were collected at 3 dpi from both *Irx1*$^{+/+}$ and *Irx1*$^{+/-}$ animals, followed by RNA extraction using miRNeasy Mini Kit (Qiagen). 3 pairs of samples (injured right side and uninjured left side) of each genotype were used for the following quality control and library preparation. Both female and male animals were used.

Following RNA quality analysis with an Agilent Technologies 2100 Bioanalyzer, Poly(A) sequencing libraries were created using the Illumina TruSeq-stranded-mRNA protocol. Two rounds of oligo-(dt) magnetic beads were used to purify adenylated mRNAs, which were then fragmented at an elevated temperature in a divalent cation buffer. Sequencing was carried out using the NovaSeq 6000 sequencing system by Illumina following library construction and quality control. The bioinformatics pipeline included: Cutadapt (removes adaptor contamination), HISAT2 (read mapping), StringTie (assembly), and then transcriptomes were merged using perl scripts and gffcompare. StringTie as well as edgeR were then used to estimate expression levels for each transcript. RNA-sequencing and analyses were performed by LC Sciences (Houston, Texas). Volcano plots were generated with VolcaNoseR and GraphPad Prism 9. GO analyses were carried out with clusterProfiler (82). Z Scores were calculated as previously described (83). Heatmaps were created with Morpheus (https://software.broadinstitute.org/morpheus).

Immunofluorescence staining and histology

Mouse mandibles were fixed in 4% paraformaldehyde (ChemCruz) and decalcified for 7-10 days in 10% EDTA. The tissues were then taken through a standard dehydration protocol and embedded in paraffin. Samples were sectioned at 7 µm using a Thermo (HM325) microtome as previously reported (84). Sections were stained using a standard H&E staining protocol. For immunofluorescence staining, slides with paraffin sections were subjected to a series of dewaxing and rehydration steps followed by citric acid antigen retrieval in a 100°C water bath for 20 minutes. Antigen retrieval steps followed a protocol described by Eckhard et al to prevent section detachment (85). The slides then were blocked with 20% donkey serum and incubated with primary antibodies overnight at 4°C. Slides were washed with 1xPBS, incubated with Alexa Fluor 488 and 594 secondary antibodies, and stained with DAPI. Confocal pictures were taken with a ZEISS 700 confocal

microscope and Zen imaging software. Images were adjusted using FIJI ImageJ software. The primary antibodies are listed in

Table **2**. Whole-mount images were taken before fixation. The tissues were then taken through dehydration, paraffin embedding, sectioning and H&E staining.

X-gal staining

Mandible and skin tissue were isolated and pre-fixed with fresh fixative buffer (2% glutaraldehyde, 100 mM NaH_2PO_4 pH=7.3, 2 mM EDTA pH=8.0, 2 mM $MgCl_2$ and 2% formaldehyde) for 1 h at room temperature. Then, tissues were washed three times (30 min each) in rinse buffer (100 mM NaH_2PO_4 pH=7.3, 1 mg/mL deoxycholic acid, 0.2% NP40 and 2 mM $MgCl_2$) at room temperature. Next, tissues were stained with X-gal solution (1 mg/mL X-gal, 5 mM $K_3Fe(CN)_6$ and 5 mM $K_4Fe(CN)_6$ dissolved in rinse buffer) at room temperature overnight. After staining, tissues were washed with 1×PBS and post-fixed in 4% paraformaldehyde for 1 h at room temperature. Tissues were then imaged with a dissection microscope in whole mount. To process stained tissue for paraffin embedding and sectioning, the previously described procedure was followed, using histo-clear in place of xylene to retain the X-gal stain. Sections were stained with eosin and imaged.

Expression and luciferase reporter constructs

To create an *Irx1* expression plasmid, the *Irx1* ORF was cloned downstream of a CMV promoter and upstream of MYC and FLAG tags as well as a polyA signal into the pLL3.7 vector backbone.

The WT mouse *Sox9* promoter (2.5kb) was cloned upstream of luciferase into the pTK-Luc vector by homology overlap using primers *Sox9 promoter F* 5'-GGTACCGAGCTCTTACGCGTGCTAGCCCGGGCTCGAGctctcgttctccaggttgtttgtg -3' and *Sox9 promoter R* 5' TGGTGGCTTTACCAACAGTACCGGAATGCCAAGCTTcgacttccagctcagggtctc -3'. To create a luciferase construct with a 4-bp mutation in the IRX1 binding site, two separate DNA fragments were amplified and simultaneously cloned into the pTK-Luc vector by homology overlap using the following primers: *Sox9 promoter Mutant Frag1F- 5'* GGTACCGAGCTCTTACGCGTG *3'; Sox9 promoter Mutant Frag1R- 5'*

aaaacaatagtcctagcattgcatgtctct *3'; Sox9 promoter Mutant Frag2F- 5'* agagacatgcaatgctaggactattgtttt *3'; Sox9 promoter Mutant Frag2R- 5'* TGGTGGCTTTACCAACAGTACCG *3'*. shRNA clones versus Irx1 were made by cloning oligos directed toward Irx1 into pSilencer 4.1. The appropriate restriction overhangs were added and cloned into BamhI and HindIII sites of the vector. Target sequence on mRNA: position: 996, forward sequence-**GCAACGAGGATGACGAGGACAA** TTCAAGAGA **TTGTCCTCGTCATCCTCGTTGC** TTTTTC; reverse sequence-GAAAAAA **GCAACGAGGATGACGAGGACAA** TCTCTTGAA **TTGTCCTCGTCATCCTCGTTGC**.

Cell culture, transfections, and reporter assays.

LS-8 (86) cells were cultured in DMEM supplemented with 10% FBS and 1% penicillin/streptomycin. *Irx1* expression plasmid and *Epgn*/*Sox9* luciferase reporter plasmid were transfected into LS-8 cells by either PEI or Lipofectamine 2000 (Invitrogen) reagents followed by real-time quantitative PCR and western blot assays. For luciferase reporter assays, cells were seeded 24h before transfection in 60mm petri dishes and transfected with 2.5ug of the expression plasmid, 5ug of reporter plasmid and 0.2ug of SV-40 β-galactosidase plasmid. Cell transfections were performed by either PEI or Lipofectamine 2000 (Invitrogen) reagents with a DNA:PEI/Lipofectamine 2000 ratio of 1:3 or 1:2. Transfected cells were incubated in 60 mm culture dishes for 48 h and fed with 10% FBS and DMEM. Following lysis with either Reporter Lysis 5X Buffer (Promega) or RIPA buffer, assays for reporter activity (luciferase assay, Promega) as well as for protein concentration (Bradford assay, Bio-Rad) were carried out. B-galactosidase was measured using the Galacto-Light Plus reagents (Tropix Inc.) as an internal normalizer. All luciferase activities were normalized to β-galactosidase activity and are shown as mean-fold differences relative to empty luciferase plasmids, and are shown as mean ± SEM. Recombinant EPGN (Prospec, Inc) was resuspended in Sterile PBS plus 10% glycerol at 0.5ug/ul and was used at 1/2000 in DMEM culture media with 10%FBS.

Lentiviral production and stable cell line establishment

HEK 293T cells are seeded in a 100 mm cell culture dish followed by PEI transfection with pMD2.G,

psPAX2 and pLL3.7 or *Irx1* expression plasmid. The medium was changed 24h post transfection and lentivirus-containing medium was collected at 48h, 72h and 96h post transfection and filtered through a 0.45 µm PVDF filter. LS-8 cells were seeded in 100 mm cell culture dishes. After 24h, the medium was replaced by lentiviral-containing medium with 8ug/mL polybrene. Medium was changed to normal culture medium after 24h. Transduced cells (GFP+) were selected through cell sorting. For cell sorting, the cells were trypsinized, washed and resuspended with PBS, and filtered through a 70 µm nylon mesh strainer. The GFP^+ cell population was sorted out through either Becton Dickinson FACS Aria II or FACS Fusion cell sorters. The sorted cells were then cultured in normal cell culture medium for stable cell line expansion.

Scratch (cell migration) assay

Scratch assays were performed on LS-8 cells or stable LS-8 expressing a control or overexpressing Irx1 lines. Cells were plated at 90% confluent density, allowed to sit down for 3 hours and then scratches were made using yellow tips. The cells were washed after the scratch and fresh culture media, or media containing 0.5ng/ul of EPGN was added. Photos were taken at the time of the scratch, 12 h and 24 h post scratch. Each condition had 4 independent wells and each well had 4 scratches. The distance of the scratch was quantitated with ImageJ and the diameter of the open scratch was expressed in um.

Cell counting assay

LS-8 or LS-8-*IRX1* cells were seeded in quadruplicates for each harvesting time points in a 35mm dish at $8x10^4$ cells/dish and cultured with DMEM (10% FBS and 1% penicillin/streptomycin). Cells were trypsinized and suspended with 1.5ml culture medium, followed by cell counting using a hemocytometer at 24h, 48h, 72h and 96h post seeding.

Real-time quantitative reverse transcription PCR (RT-qPCR)

Total RNA was isolated from cells or mouse attached gingival tissue using miRNeasy Mini Kit (Qiagen) or standard RNA preparation protocol. Reverse transcription and quantitative real-time PCR for mRNAs were

carried out by TaKaRa kit (TaKaRa, RR036A, RR420L). All Ct numbers were below 35 cycles. PCR products were examined by melting curve analysis and the sequences were confirmed. Fold changes were calculated using the $2^{-\Delta\Delta CT}$ method. The primers used for qPCR are listed in Table 3.

Western blot assays

Cell lysates from LS-8, HEK-293 cells and stable cell lines were analyzed on 4% stacking gel and 10-15% SDS-PAGE separating gels. Following electrophoresis, the protein was transferred to PVDF membrane (Millipore), immunoblotted, and detected with an HRP conjugated secondary antibody and Clarity Western ECL Blotting Substrate (Bio-Rad). The antibodies used are listed in Table 4.

Chromatin Immunoprecipitation assay (ChIP)

ChIP assays were performed as previously described (82) using the ChIP Assay Kit (Zymo research). OCCM-30 cells (an immortalized mouse cementoblast cell line expressing *Irx1 and Sox9*) were cross-linked in 1% formaldehyde at room temp for 7 minutes. Crossed linked cells were sonicated three times (6 second duration for each round, 25% of maximum amplitude) to shear the genomic DNA in to 200-1000bp fragments. Then the DNA/protein complexes were immunoprecipitated with 3ug IRX1 antibody (Sigma HPA043160) or 3ug Rabbit IgG as control. Precipitated DNAs were subjected to PCR to evaluate the enrichment of PITX2 binding. The primers used for PCR are listed in Table 3. All the PCR products were analyzed on a 1.5% agarose gel for the correct size and confirmed by sequencing. All primers are listed in the methods, text and tables.

Table 1 List of primers used for Genotyping.

Mouse strains	Genotyping primers
Irx1^{LacZNeo}	WT-F: CCGAGGCACTGAGCTGTATC<br>WT-R: TGTTCAGGTTGGAAGGGTTTCTATG<br>KO-F: CTTCAAATTGTGTCTGAGAGC<br>KO-R: GTCTGTCCTAGCTTCCTCACTG
Krt14^{CreERT}<br>(From Jax Lab)	oIMR1084: GCGGTCTGGCAGTAAAAACTATC<br>oIMR1085: GTGAAACAGCATTGCTGTCACTT<br>oIMR7338: CTAGGCCACAGAATTGAAAGATCT

Rosa26$^{Tomato-GFP}$ (From Jax Lab)	oIMR7339: GTAGGTGGAAATTCTAGCATCATCC
	oIMR7318: CTCTGCTGCCTCCTGGCTTCT
	oIMR7319: CGAGGCGGATCACAAGCAATA
	oIMR7320: TCAATGGGCGGGGGTCGTT

Table 2 List of primary antibodies and working concentration used for IF.

Antibody name	Manufacturer	Working dilution/conc.
Anti-IRX1 rabbit HPA043160	Atlas Antibodies	1:50
Anti-LacZ mouse 40-1a	DevelopmentalStudies Hybridoma Bank (DSHB)	4 µg/ml
Anti-SOX2 goat AF2018	R&D Systems	1:50
Anti-Loricrin rabbit 905101	BioLegend	1:100
Anti-E cadherin mouse 610182	BD Biosciences	1:100
Anti-KRT6A rabbit 905701	BioLegend	1:100
Anti-KRT5 rabbit ab52635	Abcam	1:50
Anti-EPGN HPA043160	Atlas Antibodies	1:50
Anti-SOX9 AB5535	Sigma-Aldrich	1:50
Anti-Krt14	Abcam	1:500
Anti-CD63-H5A4	DSHB	1:100
Anti-CD11b-H5C6	DSHB	1:100

Table 3 List of primers used for quantitative RT-PCR

Gene	Forward primer (5'-3')	Reverse primer (5'-3')
β-actin	CTCTTCCAGCCTTCCTTC	ATCTCCTTCTGCATCCTGTC
Epgn	GGGGGTTCTGATAGCAGTCTG	TCGGTGTTGTTAAATGTCCAGTT
Irx1	ACACCTGACAGCACCACCA	GCAAAAGTAAAAGATGACCCC
Ccnd2	GAGTGGGAACTGGTAGTGTTG	CGCACAGAGCGATGAAGGT
Pcna	TTTGAGGCACGCCTGATCC	GGAGACGTGAGACGAGTCCAT
Sox9	CACACGTCAAGCGACCCATGAA	TCTTCTCGCTCTCGTTCAGCAG

Table 4. List of antibodies used for western blot assays

Antibody Name	Manufacturer	Working Dilution
Anti-IRX1	Sigma	(WB) 1:2000
Anti-GAPDH mouse antibody sc32233	Santa Cruz Biotechnology	(WB) 1:10000

Table 5. List of primers used for ChIP-PCR assay.

ChIP primers	Forward primer (5'-3')	Reverse primer (5'-3')
Sox9 (IRX1)	CCTACACGTTGTGAGAATAATCGG	AACTCGTGATAGCAGCAAGCTAC

Sox9 (con)	CAACAGTAATCGTCTTTGCGCC	GAGTGGAGACTGAGAGGACTCAATC

References

82. Wu T, Hu E, Xu S, Chen M, Guo P, Dai Z, et al. clusterProfiler 4.0: A universal enrichment tool for interpreting omics data. *The Innovation.* 2021;2(3).
83. Walter W, Sánchez-Cabo F, and Ricote M. GOplot: an R package for visually combining expression data with functional analysis. *Bioinformatics.* 2015;31(17):2912-4.
84. Sun Z, Yu W, Sanz Navarro M, Sweat M, Eliason S, Sharp T, et al. Sox2 and Lef-1 interact with Pitx2 to regulate incisor development and stem cell renewal. *Development.* 2016;143(22):4115-26.
85. Eckhard AH, O'Malley JT, Nadol JB, and Adams JC. Mechanical Compression of Coverslipped Tissue Sections During Heat-induced Antigen Retrieval Prevents Section Detachment and Preserves Tissue Morphology. *Journal of Histochemistry & Cytochemistry.* 2019;67(6):441-52.
86. Chen LS, Couwenhoven RI, Hsu D, Luo W, and Snead ML. Maintenance of Amelogenin Gene Expression by Transformed Epithelial Cells of Mouse Enamel Organ. *Archs oral Biol.* 1992;37:771-8.

Supplemental Figure Legends

Suppl. Figure 1. Irx1 expression in the human oral epithelium basal stem cell layer and mesenchymal stroma tissues. A and **D**) Sections of de-identified human gingiva samples from two individuals stained with Irx1 antibody. The boxed areas are magnified areas shown in B-F. **B,E**) Irx1 expression in the basal cell layer of the oral epithelia. **C,F**) Irx1 expression in the mesenchymal stroma underlying the epithelial basal cell layer. **G**) High magnification of the cell niche in the stroma showing co-expression of Irx1 and Stro-1 in these cells.

Suppl. Figure 2. Representative human gingival samples. A and **A'**) Sections of de-identified human gingiva samples from two individuals H&E stained for morphology. **A**, 10X magnification and **A'**, 20X magnification.

Suppl. Figure 3. *Irx1* is expressed in the hair follicle bulge and dermal papilla cells of mouse skin. A) Schematics showing the structure of the mouse hair follicle. Stem cell niches are located in the bulge and DP region. **B**) X-gal and eosin staining of back skin of 2 wo *Irx1*$^{+/-}$ mice. **C**) X-gal and eosin staining of skin on the back of 6 wo *Irx1*$^{+/-}$ mice. Arrowheads indicate the X-gal staining in the upper bulge. **D**) X-gal and eosin staining of skin on the back of 15 wo *Irx1*$^{+/-}$ mice. Scale bar: 100 µm. SC niche, stem cell niche; SG, sebaceous

gland; DP, dermal papilla; HEM, hair erector muscle.

Suppl. Figure 4. CD63 levels are not affected by Irx1 expression. A,C) *Irx1*$^{+/+}$ and *Irx1*$^{+/-}$ uninjured oral tissues have low levels of CD63. **B,D**) *Irx1*$^{+/+}$ and *Irx1*$^{+/-}$ 1 dpi oral tissues have an increased CD63 immune response, but no difference between mice. Scale bar: 100µm

Suppl. Figure 5. CD11b levels are not affected by Irx1 expression. A,C) *Irx1*$^{+/+}$ and *Irx1*$^{+/-}$ uninjured oral tissues have low levels of CD11b macrophages. **B,D**) *Irx1*$^{+/+}$ and *Irx1*$^{+/-}$ 1 dpi oral tissues have an increased CD11b immune response, but no difference between mice. Scale bar: 100µm

Suppl. Figure 6. Irx1 expression increases cell proliferation. A) LS-8 oral epithelial cells that were stably over expressing Irx1 showed increased *Pcna* transcripts while *Ccnd2* transcripts were unchanged compared to empty vector (EV) cells. **B**) Western blot demonstrating Irx1 over expression (OE) and knockdown by shRNA to Irx1. **C**) Cell (LS-8) proliferation with Irx1 over expression. N=3, *p<0.05, ****p<0.0001. **D**) Relative transcript levels of *Ccna2*, *Mki67* and *Ccnd2* proliferation markers in the 3 dpi RE samples of *Irx1*$^{+/+}$ and *Irx1*$^{+/-}$ het mice. N=3, *p<0.05.

Suppl. Figure 7. Krt14 endogenous expression is unaffected in *Irx1*$^{+/-}$ mice. Krt14 expression was determined in the oral epithelium of *Irx1*$^{+/+}$ and *Irx1*$^{+/-}$ mice. Scale bar: 100µm

Suppl. Figure 8. Irx1 and Epgn regulate cell migration. A,B) LS-8 cells were transfected with plasmids expressing Irx1, sh-con (control) and sh-Irx1 (1 µg) with and without Epgn (0.5ng/ul**).** Cell monolayers were disrupted using a standard pipette to generate a uniform scratch area devoid of cells. A time course was performed to determine the amount of time required to close the open area of monolayer of cells. **C**) Quantitation of the scratch assays and effect of Irx1 and Epgn on cell migration. T0, time 0 hours; T12, time 12 hours; T24, time 24 hours. *p<0.05.

Suppl. Figure 9. Epgn regulates cell proliferation, *Sox9* and *Irx1* expression. A,B) LS-8 cells were transfected with plasmids expressing Irx1, sh-con (control) and sh-Irx1 (1 μg) with and without Epgn (0.5ng/ul**).** Cells were counted T0, T24 (after 24 hours), T48 and T72 hours. **A**) Cells transfected with Irx1 showed increased proliferation, while cells transfected with sh-Irx1 showed decreased proliferation. **B**) The addition of Epgn to the cultures rescued the decreased proliferation rates of cells expressing sh-Irx1. **C**) The addition of Epgn to cells increased the expression (transcripts) of *Sox9* and *Irx1*. N=3, *p<0.05.

Supplemental Figures

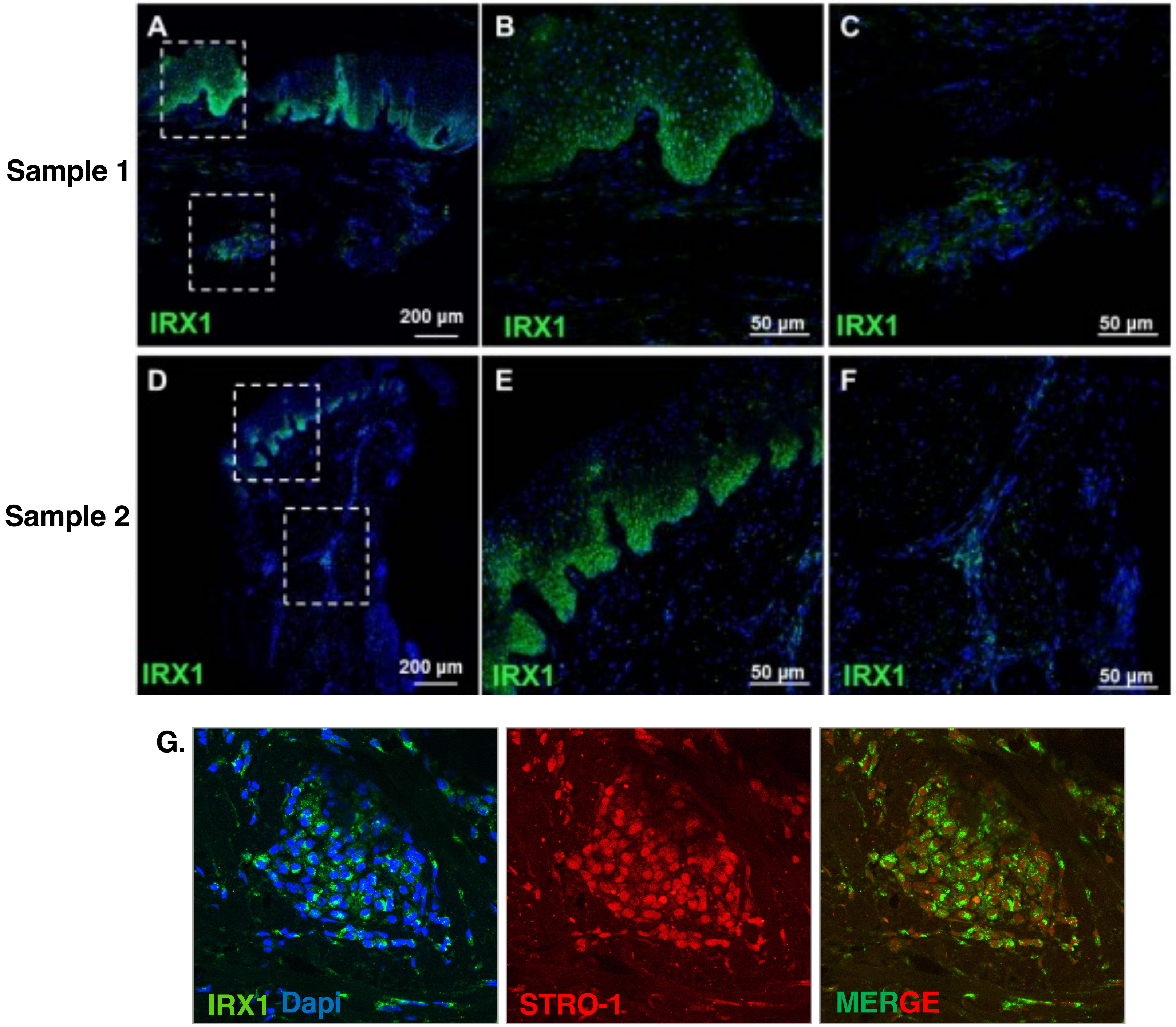
Sample 1
Sample 2
A
B
C
D
E
F
G.
IRX1
200 µm
50 µm
IRX1 Dapi
STRO-1
MERGE

Representative human gingival sample, H&E stained

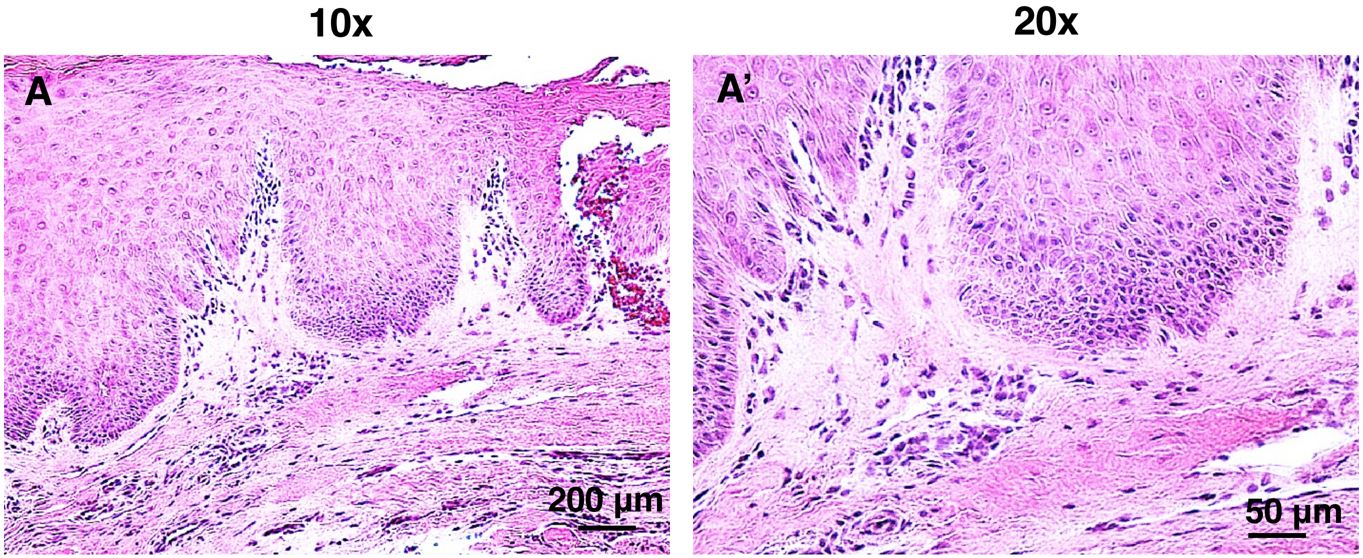

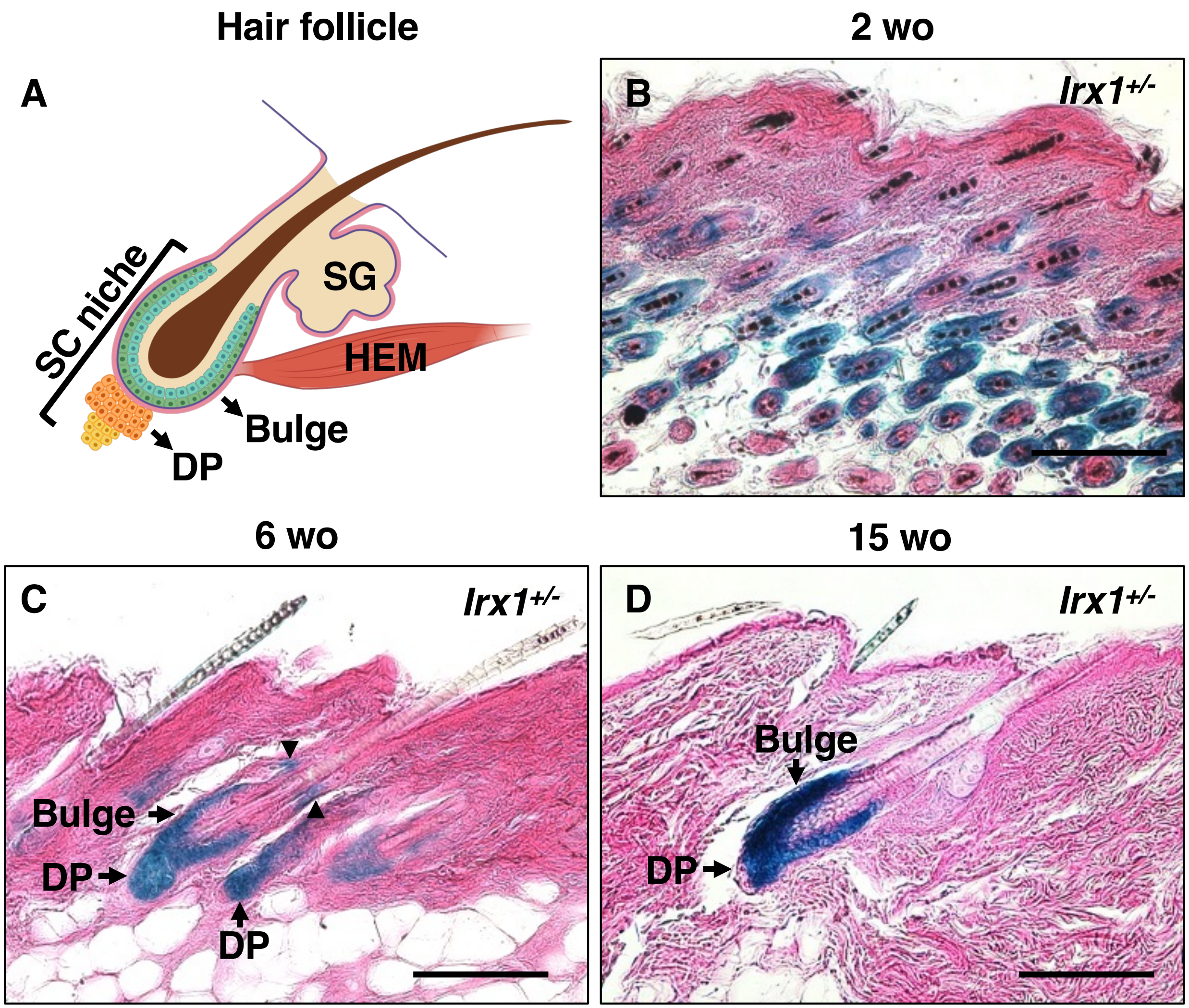
Hair follicle
2 wo
A
B
Irx1+/-
SC niche
SG
HEM
Bulge
DP
6 wo
15 wo
C
Irx1+/-
D
Irx1+/-
Bulge
DP
DP
Bulge
DP

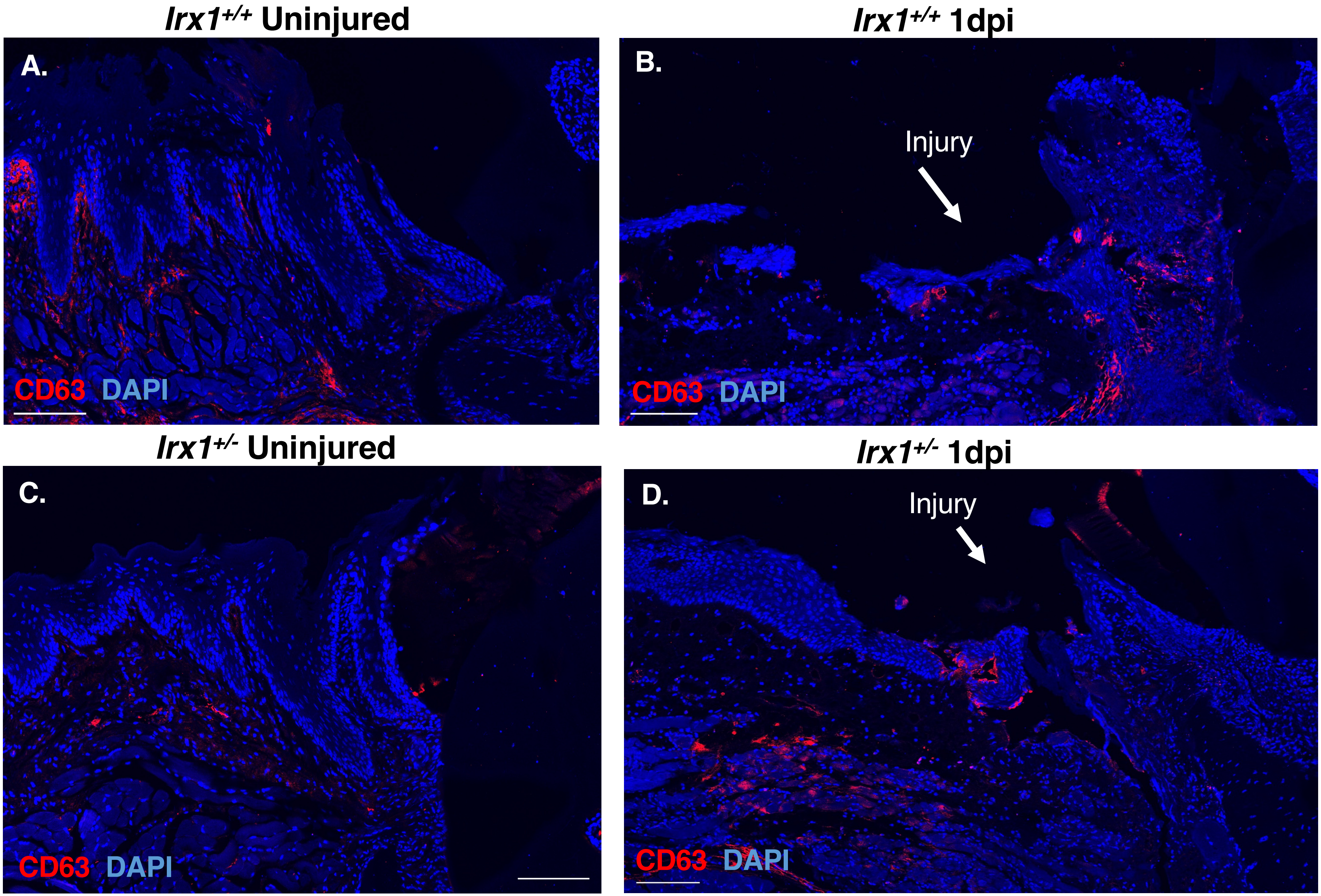
Irx1+/+ Uninjured
Irx1+/+ 1dpi
A.
CD63 DAPI
B.
Injury
CD63 DAPI
Irx1+/- Uninjured
Irx1+/- 1dpi
C.
CD63 DAPI
D.
Injury
CD63 DAPI

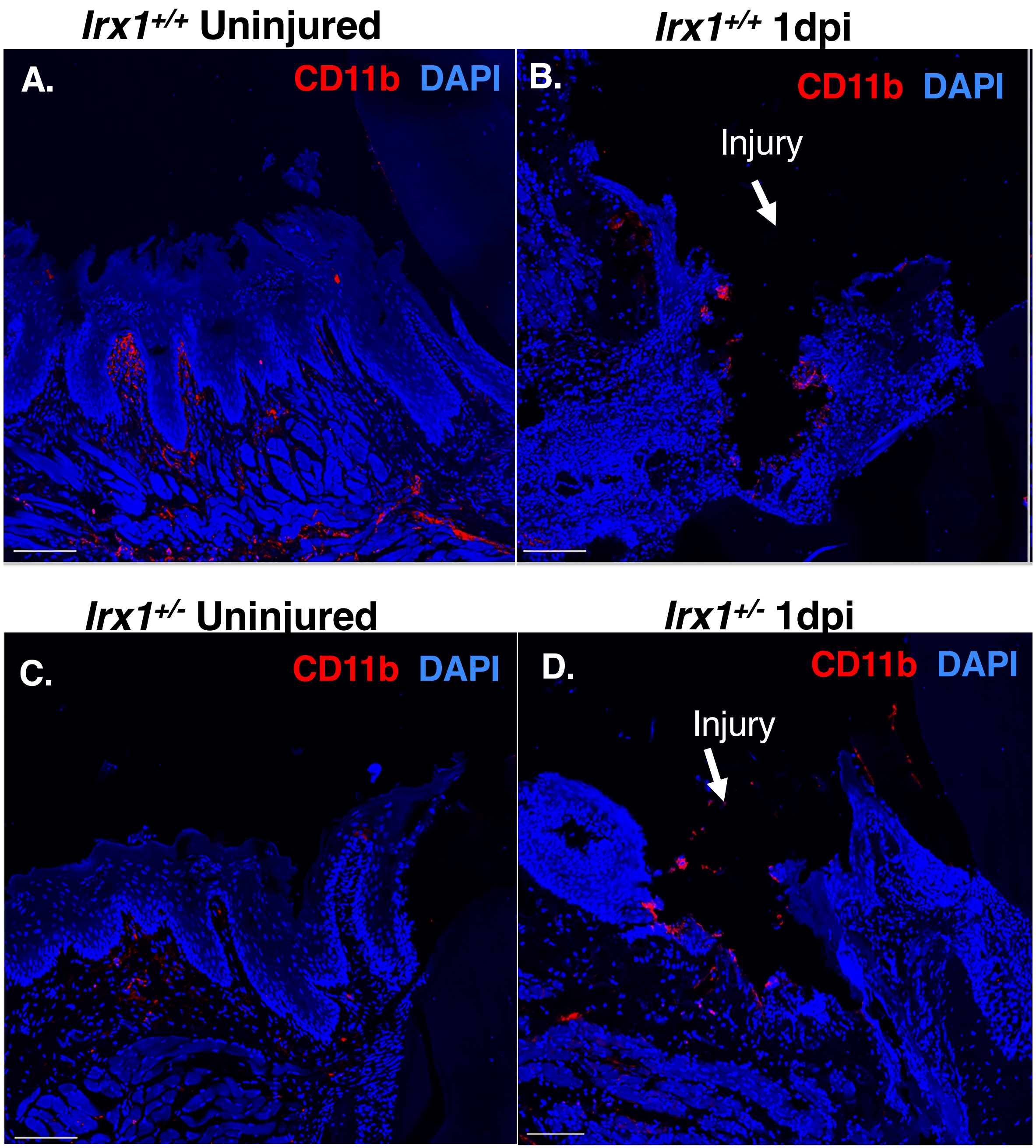
Irx1+/+ Uninjured
Irx1+/+ 1dpi
A.
CD11b DAPI
B.
CD11b DAPI
Injury
Irx1+/- Uninjured
Irx1+/- 1dpi
C.
CD11b DAPI
D.
CD11b DAPI
Injury

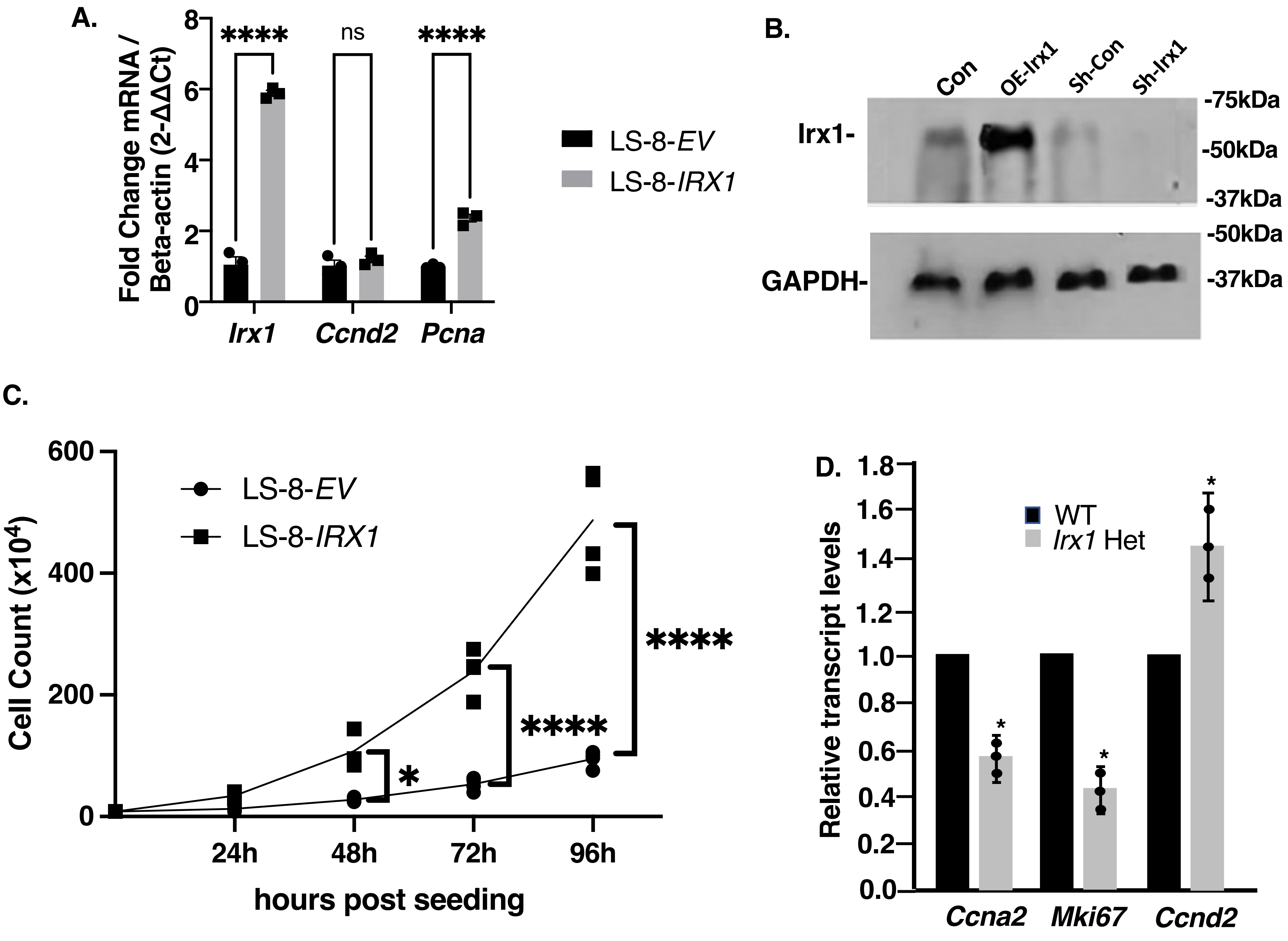
A.
Fold Change mRNA / Beta-actin (2-ΔΔCt)
8
6
4
2
0
****
ns
****
Irx1
Ccnd2
Pcna
LS-8-EV
LS-8-IRX1
B.
Con
OE-Irx1
Sh-Con
Sh-Irx1
Irx1-
GAPDH-
-75kDa
-50kDa
-37kDa
-50kDa
-37kDa
C.
Cell Count (x10^4)
600
400
200
0
LS-8-EV
LS-8-IRX1
*
****
****
24h
48h
72h
96h
hours post seeding
D.
Relative transcript levels
1.8
1.6
1.4
1.2
1.0
0.8
0.6
0.4
0.2
0.0
WT
Irx1 Het
*
*
*
Ccna2
Mki67
Ccnd2

Irx1$^{+/+}$ **Uninjured**

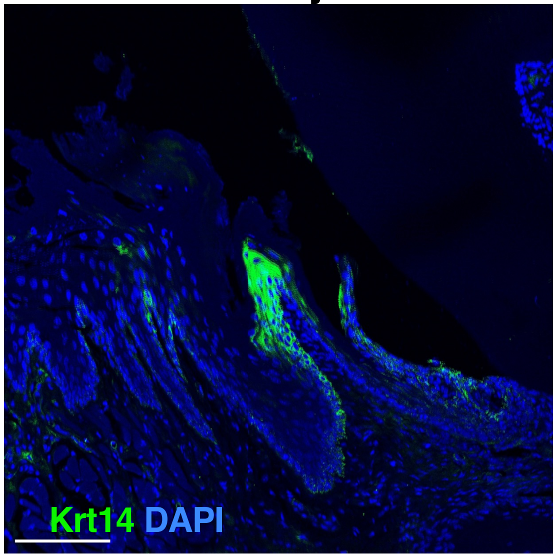

Irx1$^{+/-}$ **Uninjured**

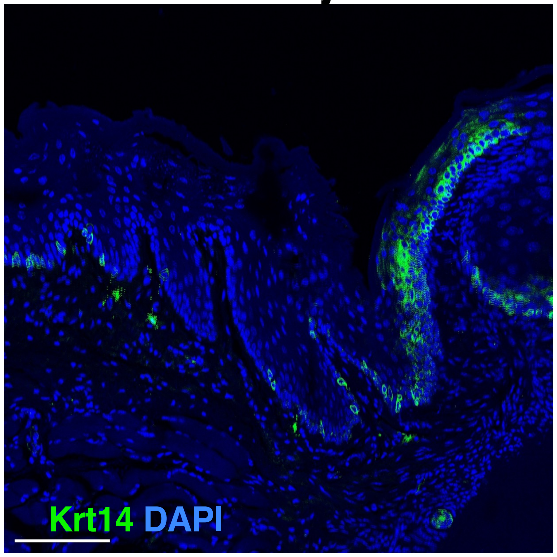

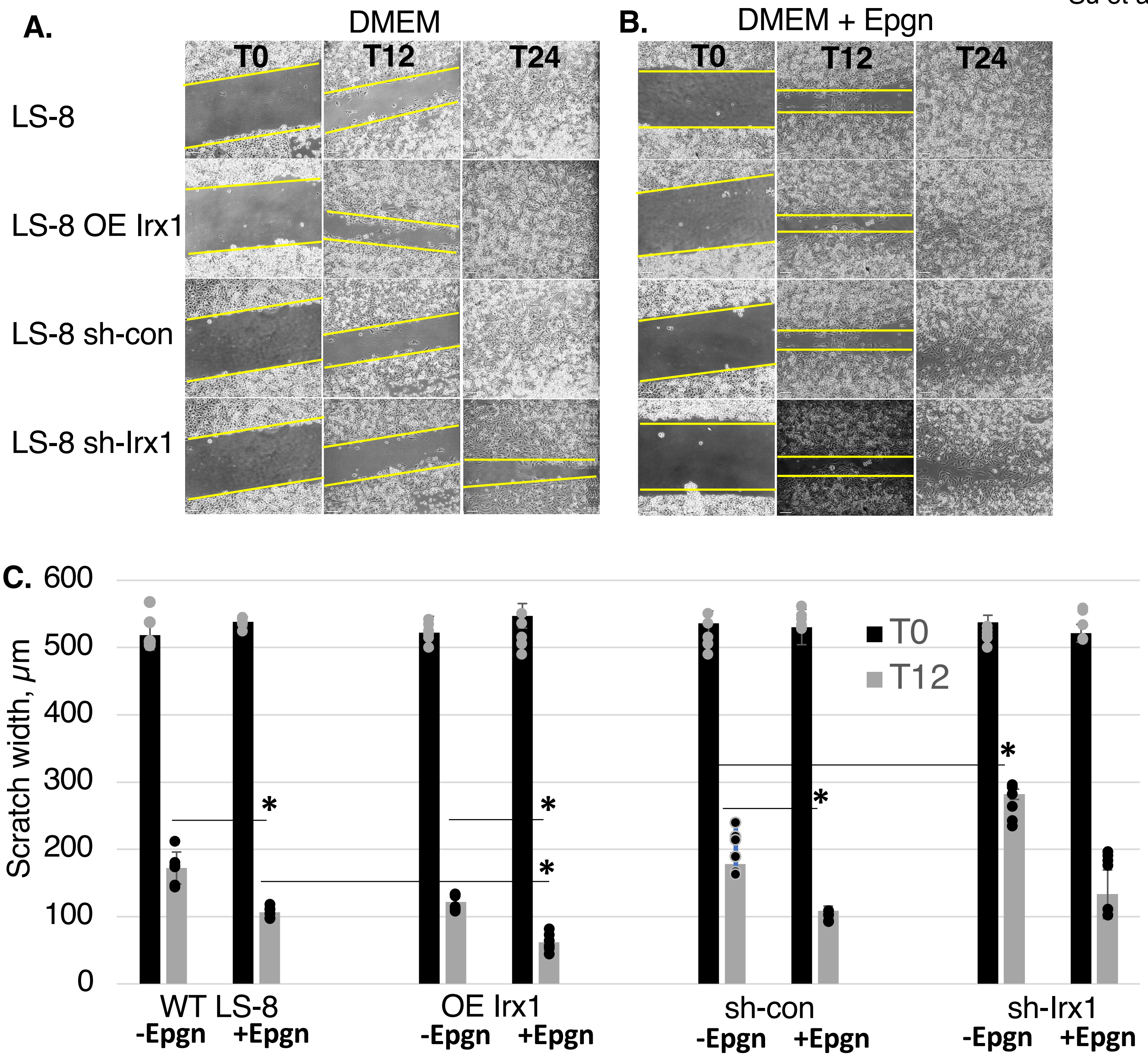
A.
DMEM
T0
T12
T24
LS-8
LS-8 OE Irx1
LS-8 sh-con
LS-8 sh-Irx1
B.
DMEM + Epgn
T0
T12
T24
C.
Scratch width, μm
600
500
400
300
200
100
0
T0
T12
*
WT LS-8
-Epgn +Epgn
OE Irx1
-Epgn +Epgn
sh-con
-Epgn +Epgn
sh-Irx1
-Epgn +Epgn

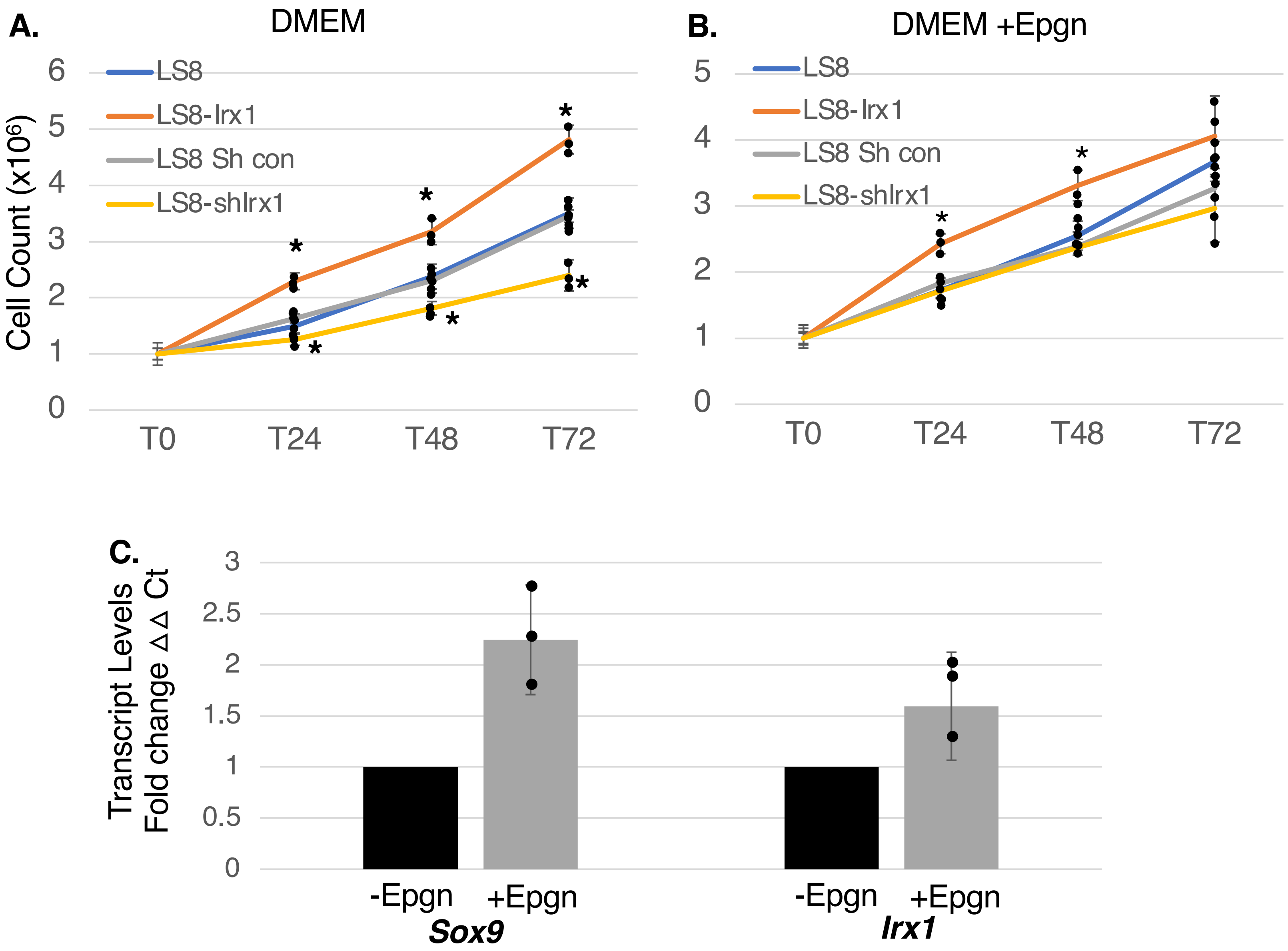
A.
DMEM
LS8
LS8-Irx1
LS8 Sh con
LS8-shIrx1
Cell Count (x10⁶)
0
1
2
3
4
5
6
T0
T24
T48
T72
*
B.
DMEM +Epgn
LS8
LS8-Irx1
LS8 Sh con
LS8-shIrx1
0
1
2
3
4
5
T0
T24
T48
T72
*
C.
Transcript Levels
Fold change ΔΔ Ct
0
0.5
1
1.5
2
2.5
3
-Epgn
+Epgn
Sox9
-Epgn
+Epgn
Irx1
