# Supplementary figures and images for: Irx1 mechanisms for oral epithelial basal stem cell plasticity during reepithelialization after injury
Source: JCI Insight. 2025 Jan 9;10(1):e179815. doi: 10.1172/jci.insight.179815 (PMC11721312; doi:10.1172/jci.insight.179815)

# Irx1 Western Blots from 5-14-24

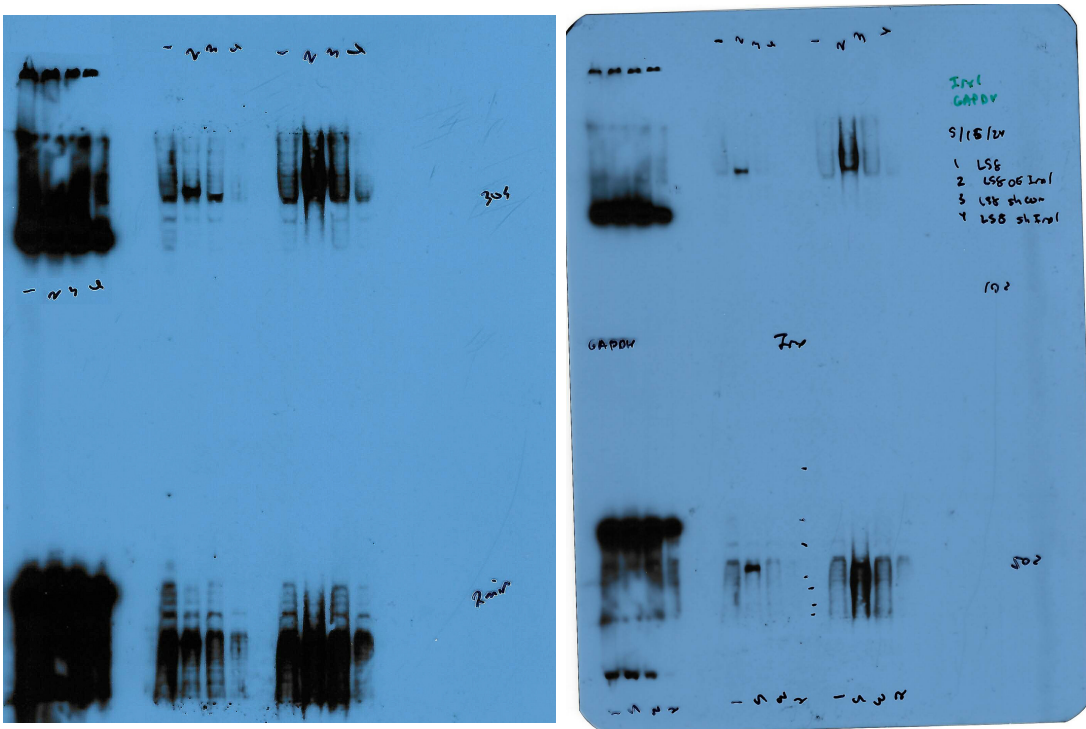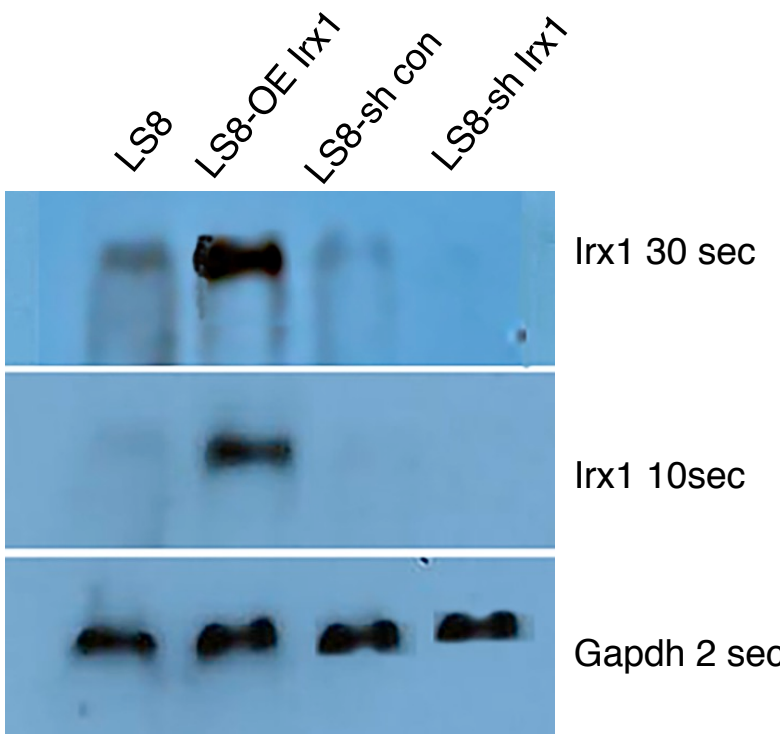

Supplement: Unedited blot and gel images [file jciinsight-10-179815-s234.pdf]
